# An evaluation of variation in published estimates of schizophrenia prevalence from 1990─2013: a systematic literature review

**DOI:** 10.1186/s12888-015-0578-7

**Published:** 2015-08-12

**Authors:** Jason C. Simeone, Alexandra J. Ward, Philip Rotella, Jenna Collins, Ricarda Windisch

**Affiliations:** 1 Evidera, 430 Bedford Street, Suite 300, Lexington, MA 02420 USA; 2 F. Hoffmann-La Roche Ltd, Basel, Switzerland

## Abstract

**Background:**

There is a lack of consistency in findings across studies on the prevalence of schizophrenia, and no recent systematic review of the literature exists. The purpose of this study is to provide an updated systematic review of population-based prevalence estimates and to understand the factors that could account for this variation in prevalence estimates.

**Methods:**

MEDLINE, Embase, and PsycInfo databases were searched for observational studies describing schizophrenia prevalence in general populations from 2003–2013 and supplemented by studies from a prior review covering 1990–2002. Studies reporting prevalence estimates from specialized populations such as institutionalized, homeless, or incarcerated persons were excluded. Prevalence estimates were compared both across and within studies by factors that might contribute to variability using descriptive statistics.

**Results:**

Sixty-five primary studies were included; thirty-one (48 %) were from Europe and 35 (54 %) were conducted in samples of ≥50,000 persons. Among 21 studies reporting 12-month prevalence, the median estimate was 0.33 % with an interquartile range (IQR) of 0.26 %–0.51 %. The median estimate of lifetime prevalence among 29 studies was 0.48 % (IQR: 0.34 %–0.85 %). Prevalence across studies appeared to vary by study design, geographic region, time of assessment, and study quality scores; associations between study sample size and prevalence were not observed. Within studies, age-adjusted estimates were higher than crude estimates by 17 %–138 %, the use of a broader definition of schizophrenia spectrum disorders compared to schizophrenia increased case identification by 18 %–90 %, identification of cases from inpatient-only settings versus any setting decreased prevalence by 60 %, and no consistent trends were noted by differing diagnostic criteria.

**Conclusions:**

This review provides updated information on the epidemiology of schizophrenia in general populations, which is vital information for many stakeholders. Study characteristics appear to play an important role in the variation between estimates. Overall, the evidence is still sparse; for many countries no new studies were identified.

**Electronic supplementary material:**

The online version of this article (doi:10.1186/s12888-015-0578-7) contains supplementary material, which is available to authorized users.

## Background

Schizophrenia is a serious, complex brain disorder, with a reported median incidence of 15.2 per 100,000 persons [1] and a pooled lifetime prevalence of 0.40 % (10 %–90 % quantiles: 0.16–1.21 %), both estimates being based on a review by Saha et al. [2] No comprehensive review has followed Saha et al.’s systematic search in 2003. Moreover, prior reviews highlight the variability in schizophrenia prevalence estimates [3–6]. Eaton, for example, noted a 12-fold variation in point prevalence and a 10-fold variation in lifetime prevalence, [3] while Goldner et al. observed a 13-fold variation in lifetime prevalence of schizophrenia [6].

Inherent variability between estimates may in part be due to the heterogeneity and complexity of the disease [1]. However, other factors also likely contribute to variation observed between reported prevalence estimates. Study design (e.g. cohort or cross-sectional study) and methods can affect case ascertainment in an epidemiological study [7, 8]. Population and health care system differences exist at the national and regional level, which highlights the importance of recording the geographic region of an epidemiological study. The sample size of the overall population can be an indicator of the generalizability of an estimate and outlier estimates may be reported from very small populations [9]. Factors such as the diagnostic criteria for schizophrenia have changed over time: The Diagnostic and Statistical Manual of Mental Disorders (DSM-V) currently guides physicians to diagnose schizophrenia along a continuum of severity, from the less severe delusional disorder to the more severe schizoaffective disorder [10]. Therefore, the period in which a study was conducted may influence the number of cases identified and the resulting prevalence estimate. Other factors such as study setting (e.g., prisons, hospitals, or the general community) also likely contribute to this variability [1, 2, 11].

According to McGrath, the variability between estimates requires the use of systematic reviews and—in a second step—pooled estimates [1]. However, pooled estimates mask essential information when variability in estimates is mainly due to factors such as differences in study design or populations, and a better understanding may be gained from looking at these studies without pooling estimates. The objective of this review is two-fold: 1) to provide an updated systematic review of population-based prevalence estimates; and 2) to understand all main factors that could account for variability in published prevalence estimates, including study design, geographic region, sample size, study dates, and study quality.

## Methods

This systematic review adheres to current best practices for conducting systematic reviews of the literature [12, 13]. The data source was literature published from January 1, 2003 to October 9, 2013, and the methods used to perform this review involved both electronic and manual components. Studies were identified from the literature by searching the MEDLINE (via PubMed), Embase, and PsycINFO databases for the terms “schizophrenia” and “prevalence”. Searches were limited to studies with human subjects and published in the English language. Case reports, letters, commentaries, editorials, reviews, clinical trials, reviews, and *in vitro* studies were excluded. These three electronic searches were supplemented by additional targeted electronic searches (using broader schizophrenia/psychosis terms) and by a manual search of the bibliographies of all accepted studies. Search results from the various sources were combined, and the duplicate records were removed. The titles and abstracts of each citation were screened and the full text of each potentially relevant citation was retrieved and reviewed. Studies identified in the systematic literature review conducted by Saha et al. [2] were also screened for inclusion in our review if they were published from 1990–2002.

Population-based observational studies (retrospective or prospective) reporting on the prevalence of schizophrenia in the general population were selected for this review. To minimize variation caused by study setting, studies performed in high-risk or other sub-populations (e.g., institutionalized, incarcerated, homeless subjects) were excluded. Studies with fewer than 200 screened people were also excluded to minimize outlier estimates resulting from small sample sizes.

Both descriptive and quantitative study- and patient-level data from accepted studies were extracted into a data extraction form by a single investigator and then reviewed against the original study by a second investigator. Quantitative data included prevalence estimates which were extracted as reported in each study and then standardized to percentages to facilitate comparisons between studies. Study country was classified by region as presented in Additional file 1: Table S1. The level of evidence score (see Additional file 1: Table S2) was adapted from the review by Saha and colleagues [2]. The maximum score was 15 points per study, and a higher score indicated a greater level of evidence.

Occasionally studies presented multiple prevalence estimates per period (e.g. 12 months, lifetime); for example, a study may have presented three estimates of lifetime prevalence that were calculated using three different sets of diagnostic criteria. In cases such as these, only one estimate was selected per period using the following pre-specified criteria, which were developed to minimize variability between estimates for comparative purposes. The criteria involved selecting 1) crude estimates preferentially, with adjusted estimates only selected if no crude estimates were available; 2) the most recent estimate; 3) an estimate from the most broad catchment area; 4) the most broad case ascertainment method (e.g., cases identified from inpatient, outpatient, and emergency room visits, rather than just one setting); 5) the most recent diagnostic criteria; and 6) estimates based on a narrow definition of schizophrenia, when estimates derived from more expansive definitions were presented. Therefore, studies contributed a maximum of one prevalence estimate per period for the purposes of these analyses, and estimates from different time periods were not compared.

Descriptive statistics, including means, standard deviations, median, ranges, and interquartile ranges (IQRs) were used to summarize prevalence estimates and other continuous variables. Categorical variables such as study characteristics were summarized using counts and proportions. Sub-group analyses of factors including study design, geographic region, sample size, study dates, and quality score were conducted separately for 12-month and lifetime prevalence estimates. To compare estimates from the same prevalence periods, this review emphasizes 12-month and lifetime prevalence estimates (the most commonly reported periods). However, point prevalence and estimates from other periods are also briefly summarized for comprehensiveness. Other factors were also assessed within studies when possible, including differences between prevalence periods, various methods of case identification, and temporal trends.

## Results

### Study selection

**Fig. 1:**
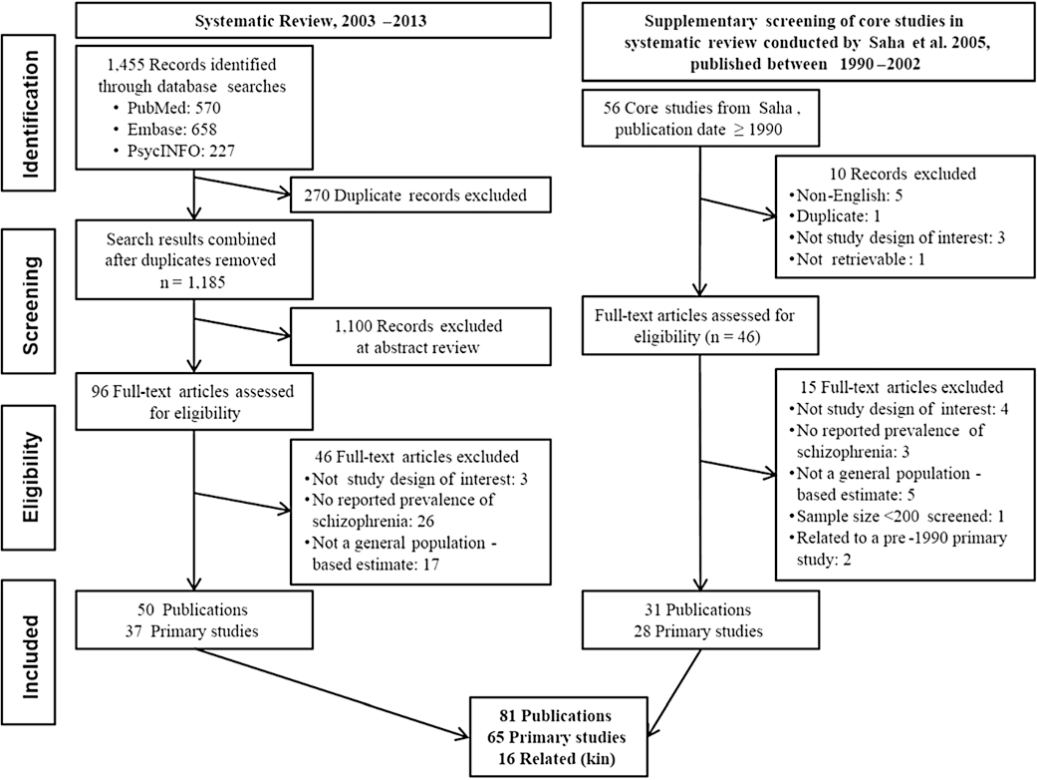
Flow diagram of study selection

A total of 1185 unique citations were identified from MEDLINE, Embase, and PsycINFO in the systematic review (Fig. 1). At the abstract screening level, 1100 citations were excluded for the following reasons: prevalence of schizophrenia not reported (n = 802), study type (n = 238), and not a sample from the general population (n = 60). Eighty-five full-text articles were retrieved, plus two articles identified from the targeted searches, and nine identified from manual bibliography checks. Thirty-seven primary studies and 13 related publications (e.g. a different prevalence study published by the same investigators in a particular catchment area and overlapping time period) were included from the 2003–2013 systematic literature review.

Of the 142 articles identified in the Saha review, [2] 56 were published from 1990–2002 and retrieved for further screening. The year 1990 was used as a cut-off to limit the search to more current studies, and this date was selected after verifying that no major studies were excluded prior to that date. Twenty-eight primary articles and 3 related publications from Saha et al. met the inclusion criteria of this review (with the main differences being a restriction to observational studies on the general population published in English), as presented in Fig. 1.

In total, 65 primary studies [14–78] and 16 related publications [79–94] published from 1990–2013 were identified for inclusion in this review.

### Study characteristics

Among the studies included in this review, 29 were from Europe, 13 were from Asia, 10 were from North America, eight were from Africa, four were from Oceania, and one was a multinational study reporting country-specific estimates for 52 countries from all regions (Table 1). Over half of the studies (35 or 53.8 %) were conducted with sample sizes of 50,000 people or greater. Study design was evenly split between cross-sectional studies (50.8 %) and cohort studies (49.2 %). The cohort studies primarily utilized healthcare databases or case registers (n = 25), though also included five birth cohort studies [16, 24, 38, 67, 70] and two follow-up studies of previously defined cohorts [72, 73]. Although publication dates ranged from 1990–2013, over half of the studies (55.4 %) described samples recruited prior to 1999. Forty studies (61.5 %) reported prevalence among diagnosed populations, and the age of patients sampled in each study varied widely, from narrow ranges such as 15–38 years [24] to no age restrictions. The mean quality score and corresponding standard deviation was 8.8 ± 2.6 across all studies, ranging by region from 7.7 among studies conducted in North America to 10.1 among studies conducted in Africa.

**Table 1:** Characteristics of the 65 included studies

Characteristic	Europe	Asia	North America	Africa	Oceania	All Regions
Number of studies^a^	29	13	10	8	4	65
Sample size						
<5000	3 (10.3 %)	1 (7.7 %)	0	2 (25.0 %)	1 (25.0 %)	7 (10.8 %)
5000–9999	3 (10.3 %)	4 (30.8 %)	1 (10.0 %)	0	1 (25.0 %)	9 (13.8 %)
10,000–49,999	6 (20.7 %)	2 (15.4 %)	1 (10.0 %)	4 (50.0 %)	1 (25.0 %)	14 (21.5 %)
50,000–99,999	4 (13.8 %)	1 (7.7 %)	0	2 (25.0 %)	0	7 (10.8 %)
100,000+	13 (44.8 %)	5 (38.5 %)	8 (80.0 %)	0	1 (25.0 %)	28 (43.1 %)
Study design						
Cohort	16 (55.2 %)	4 (30.8 %)	8 (80.0 %)	0	4 (100 %)	32 (49.2 %)
Cross-sectional	13 (44.8 %)	9 (69.2 %)	2 (20.0 %)	8 (100 %)	0	33 (50.8 %)
Publication date						
1990–2002	17 (58.6 %)	2 (15.4 %)	2 (20.0 %)	5 (62.5 %)	2 (50.0 %)	28 (43.1 %)
2003–2013	12 (41.4 %)	11 (84.6 %)	8 (80.0 %)	3 (37.5 %)	2 (50.0 %)	37 (56.9 %)
Assessment year^b^						
<1990	10 (34.5 %)	1 (7.7 %)	0	2 (25.0 %)	0	13 (20.0 %)
1990–1999	10 (34.5 %)	3 (23.1 %)	4 (40.0 %)	4 (50.0 %)	2 (50.0 %)	23 (35.4 %)
2000–2009	7 (24.1 %)	9 (69.2 %)	6 (60.0 %)	1 (12.5 %)	1 (25.0 %)	25 (38.5 %)
Not reported	2 (6.9 %)	0	0	1 (12.5 %)	1 (25.0 %)	4 (6.2 %)
Prevalence period type^c^						
Point	6 (20.7 %)	4 (30.8 %)	1 (10.0 %)	2 (25.0 %)	1 (25.0 %)	14 (21.5 %)
12 months	11 (37.9 %)	4 (30.8 %)	5 (50.0 %)	1 (12.5 %)	1 (25.0 %)	22 (33.8 %)
Other period	7 (24.1 %)	2 (15.4 %)	2 (20.0 %)	2 (25.0 %)	0	13 (20.0 %)
Lifetime	13 (44.8 %)	6 (46.2 %)	3 (30.0 %)	5 (62.5 %)	2 (50.0 %)	30 (46.2 %)
Quality score						
Mean ± SD	9.0 ± 2.6	8.8 ± 3.4	7.7 ± 2.1	10.1 ± 1.6	8.8 ± 1.5	8.8 ± 2.6

^a^One study (Nuevo et al.) reported on multiple regions, and is counted only in the “All regions” category. Therefore, the number of studies in each region sum to only 64 studies

^b^For studies that assessed prevalence over a range of years, the most recent year was used

^c^Multiple types of prevalence could be reported within the same study; therefore, percents do not add up to 100

### 12-month prevalence

**Fig. 2:**
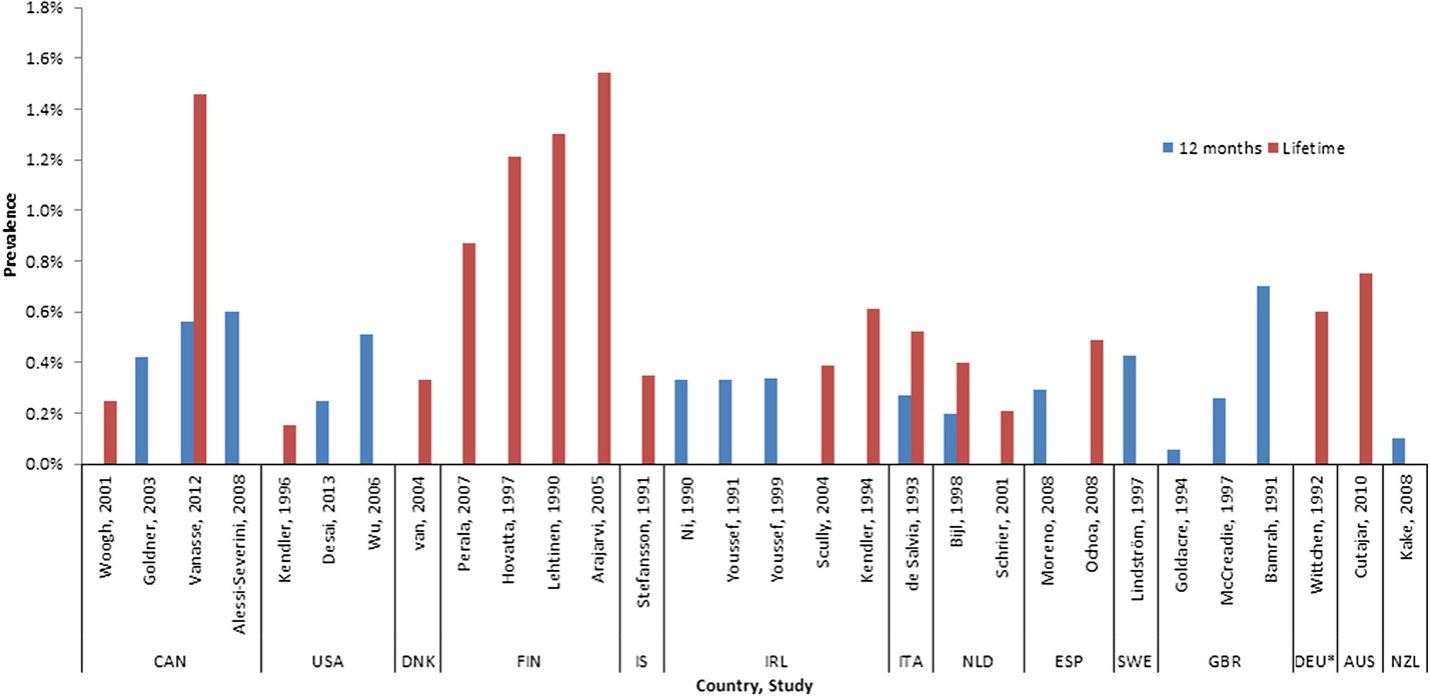
Prevalence of Schizophrenia in North America, Europe, Australia, and New Zealand. Countries are designated by 3-letter International Organization for Standardization (ISO) code. CAN: Canada; USA: United States; DNK: Denmark; FIN: Finland; IS: Iceland; IRL: Ireland; ITA: Italy; NLD: Netherlands; ESP: Spain; SWE: Sweden; GBR: United Kingdom; DEU: Germany; AUS: Australia; NZL: New Zealand. *Former West Germany

Twenty-two studies reported 12-month prevalence of schizophrenia. However, one study [34] reported prevalence in the UK in 2005 to be 74.7 per 1000 person-years-exposure, which could not be standardized to a percentage. Therefore, only 21 articles are represented in Tables 2 and 3. The median estimate across all studies was 0.33 % (range: 0.06 %–0.75 %; IQR: 0.26 %–0.51 %, Table 3). Across geographic regions, the median 12-month prevalence was 0.31 % in Europe (IQR: 0.26 %–0.34 %) and 0.51 % in North America (IQR: 0.42 %–0.56 %). Only one study each reported 12-month prevalence from Oceania (0.10 %) and Africa (0.75 %). Studies (n = 16) that reported 12-month prevalence estimates in developed countries in Europe (n = 10), North America (n = 5), and Oceania (n = 1) are presented in Fig. 2.

**Table 2:** 12-month period prevalence estimates, by country

Author, Year	Country	Sample size	Prevalence estimate	Study design	Screened age range
Africa					
Jay, 1997	Reunion Island (France)	88,000	0.75 %	Cross-sectional	15+
North America					
Goldner, 2003	Canada	2,703,588	0.42 %	Cohort	15–64
Vanasse, 2012	Canada	5,996,925	0.56 %	Cohort	18+
Alessi-Severini, 2008	Canada	2,703,588	0.6 %	Cohort	All ages
Desai, 2013	US	140,000	0.25 %	Cohort	All ages
Wu, 2006	US	10,000,000	0.51 %^a^	Cohort	All ages
Asia					
Cho, 2010	South Korea	6,510	0.1 %^a^	Cross-sectional	18–64
Chang, 2008	South Korea	40,000,000	0.4 %	Cohort	All ages
Chien, 2004	Taiwan	136,045	0.33 %	Cohort	15+
Chien, 2009	Taiwan	4,417	0.58 %	Cohort	18+
Europe					
Ni Nuallain, 1990	Ireland	112,000	0.33 %	Cohort	15–64
Youssef, 1991	Ireland	25,178	0.33 %	Cross-sectional	All ages
Youssef, 1999	Ireland	21,520	0.34 %	Cross-sectional	All ages
de Salvia, 1993	Italy	72,512	0.27 %	Cohort	15+
Bijl, 1998	Netherlands	7,076	0.2 %^a^	Cross-sectional	18–64
Moreno, 2008	Spain	400,000	0.29 %^a^	Cohort	14+
Lindström, 1997	Sweden	64,886	0.43 %	Cohort	>18
Goldacre, 1994	UK	527,000	0.06 %	Cohort	All ages
McCreadie, 1997	UK	140,603	0.26 %	Cross-sectional	All ages
Bamrah, 1991	UK	74,176	0.70 %	Cohort	15+
Oceania					
Kake, 2008	New Zealand	3,736,269	0.10 %	Cohort	All ages

^a^Adjusted prevalence

**Table 3:** Summary of 12-month period prevalence estimates

Strata	N	Min	IQ25	Median	IQ75	Max
Overall	21	0.06 %	0.26 %	0.33 %	0.51 %	0.75 %
Design						
Cross-sectional	6	0.10 %	0.22 %	0.30 %	0.34 %	0.75 %
Cohort	15	0.06 %	0.28 %	0.40 %	0.54 %	0.70 %
Region						
Africa	1	0.75 %	0.75 %	0.75 %	0.75 %	0.75 %
North America	5	0.25 %	0.42 %	0.51 %	0.56 %	0.60 %
Asia	4	0.10 %	0.27 %	0.37 %	0.45 %	0.58 %
Europe	10	0.06 %	0.26 %	0.31 %	0.34 %	0.70 %
Oceania	1	0.10 %	0.10 %	0.10 %	0.10 %	0.10 %
Sample Size						
<5000	1	0.58 %	0.58 %	0.58 %	0.58 %	0.58 %
5000–9999	2	0.10 %	0.13 %	0.15 %	0.18 %	0.20 %
10,000–49,999	2	0.33 %	0.33 %	0.34 %	0.34 %	0.34 %
50,000–99,999	4	0.27 %	0.39 %	0.57 %	0.71 %	0.75 %
100,000+	12	0.06 %	0.26 %	0.33 %	0.44 %	0.60 %
Publication Date						
1990–2002	10	0.06 %	0.26 %	0.33 %	0.41 %	0.75 %
2003–2013	11	0.10 %	0.27 %	0.40 %	0.54 %	0.60 %
Assessment Year^a^						
<1990	6	0.06 %	0.29 %	0.33 %	0.61 %	0.75 %
1990–1999	7	0.20 %	0.28 %	0.33 %	0.38 %	0.43 %
2000–2009	8	0.10 %	0.21 %	0.46 %	0.57 %	0.60 %
Quality Score						
0–7	7	0.06 %	0.33 %	0.42 %	0.55 %	0.60 %
8–10	10	0.10 %	0.26 %	0.31 %	0.53 %	0.75 %
11–15	4	0.10 %	0.27 %	0.33 %	0.33 %	0.34 %

One study (McCreadie et al.) utilized database methods for one region and cross-sectional methods for the other 2 regions studied; the 3 regional estimates were pooled and this study has been categorized as “cross-sectional” for these analyses

^a^Selected estimates are the most recent year available. When assessment spanned multiple years, the median year was considered. 4 estimates did not have assessment years reported

The median 12-month prevalence estimate from cohort studies (0.40 %) was higher than the median estimate from cross-sectional studies (0.30 %). While the median prevalence was 0.33 % for samples assessed from <1990 (IQR: 0.29 %–0.61 %) and 1990–1999 (IQR: 0.28 %–0.38 %), the median prevalence was 0.46 % among eight studies that assessed samples from 2000–2009 (IQR: 0.21 %–0.57 %). The median prevalence for studies with a score of 0–7 was 0.42 % (IQR: 0.33 %–0.55 %), decreasing to 0.31 % (IQR: 0.26 %–0.53 %) among studies with a score of 8–10, and to 0.33 % (IQR: 0.27 %–0.33 %) among studies with a score of 11–15. No clear pattern related to 12-month prevalence by study sample size was observed.

### Lifetime prevalence

Thirty studies reported lifetime prevalence estimates (Table 4). The overall median lifetime prevalence estimate across the studies included in this review was 0.48 % (range: 0.06 %–5.00 %; IQR: 0.34 %–0.85 %). Among the studied regions, the lowest reported median lifetime prevalence was in North America (0.25 %; IQR: 0.20 %–0.86 %), while two studies from Oceania reported prevalence estimates of 0.40 % and 0.75 %. Thirteen studies were from Europe, with a median lifetime prevalence of 0.52 % (IQR: 0.39 %–0.87 %). Fig. 2 presents all estimates.

**Table 4:** Lifetime prevalence estimates, by country

Author, Year	Country	Sample size	Prevalence estimate	Study design	Screened age range
Africa					
Kebede, 1999	Ethiopia	10,203	0.4 %^a^	Cross-sectional	15+
Kebede, 2003	Ethiopia	68,378	0.47 %	Cross-sectional	15–49
Awas, 1999	Ethiopia	10,468	0.8 %^a^	Cross-sectional	15+
Rumble, 1996	South Africa	3,032	5 %^a^	Cross-sectional	NR
Bondestam, 1990	Tanzania	10,776	0.06 %	Cross-sectional	All ages
North America					
Woogh, 2001	Canada	140,000	0.25 %	Cohort	NR
Vanasse, 2012	Canada	5,996,925	1.46 %	Cohort	18+
Kendler, 1994	US	8,098	0.15 %^a^	Cross-sectional	15–54
Asia					
Chen, 1993	China	7,229	0.13 %	Cross-sectional	18–64
Ran, 2003	China	123,572	0.41 %	Cross-sectional	15+
Xiang, 2008	China	5,926	0.52 %	Cross-sectional	15+
Barrett, 2005	Malaysia	91,056	0.24 %	Cross-sectional	NR
Cho, 2010	South Korea	6,510	0.1 %^a^	Cross-sectional	18–64
Phanthunane, 2010	Thailand	11,700	0.88 %^a^	Cross-sectional	15–59
Europe					
van, 2004	Denmark	1,020,063	0.33 %	Cohort	25
Perala, 2007	Finland	8,028	0.87 %^a^	Cross-sectional	30+
Hovatta, 1997	Finland	2,400,000	1.21 %	Cohort	5–51
Lehtinen, 1990	Finland	7,217	1.3 %^a^	Cross-sectional	30+
Arajarvi, 2005	Finland	12,368	1.54 %	Cohort	29–58
Wittchen, 1992	Germany	1,366	0.60 %^a^	Cohort	25–64
Stefansson, 1991	Iceland	862	0.35 %	Cohort	55–57
Scully, 2004	Ireland	29,542	0.39 %	Cross-sectional	All ages
Kendler, 1994	Ireland	20,686	0.61 %	Cohort	15–57
de Salvia, 1993	Italy	72,512	0.52 %	Cohort	15+
Schrier, 2001	Netherlands	337,362	0.21 %	Cohort	20–64
Bijl, 1998	Netherlands	7,076	0.4 %^a^	Cross-sectional	18–64
Ochoa, 2008	Spain	1,645	0.49 %	Cross-sectional	18+
Oceania					
Cutajar, 2010	Australia	2,677	0.75 %	Cohort	18–58
Waldo, 1999	Micronesia	5,500	0.40 %	Cohort	All ages
All Regions					
Nuevo, 2012	Multinational (52 countries)	181,445	1.11 %^a^	Cross-sectional	18+

^a^Adjusted prevalence

The median lifetime prevalence was lower for cross-sectional studies (0.44 %; IQR: 0.28 %–0.85 %) than cohort studies (0.56 %; IQR: 0.35 %–0.87 %). The median lifetime prevalence from assessment periods prior to 1990 (0.44 %; IQR: 0.26 %–0.61 %) was similar to that from assessment periods between 1990 and 1999 (0.40 %; IQR: 0.32 %–0.61 %); median prevalence was highest among studies with assessment periods from 2000–2009 (0.70 %; 0.49 %–0.94 %). The median lifetime prevalence was highest among studies with quality scores from 0–7 (0.75 %; IQR: 0.23 %–1.00 %) when compared to studies with quality scores from 8–10 (0.45; IQR: 0.35 %–0.59 %) and studies with quality scores from 11–15 (0.47 %; IQR: 0.39 %–0.80 %). No trends could be discerned regarding sample size and median lifetime prevalence.

One study, published by Nuevo and colleagues, presented the results of the World Health Organization’s 2003 World Health Survey (WHS) and detailed estimates of schizophrenia prevalence across 52 countries [56]. Household respondents aged 18+ completed a standardized questionnaire that collected data on demographics, self-reported diagnoses, and treatment of schizophrenia and psychotic symptoms, and the results were considered to be nationally representative. In this study, lifetime prevalence estimates varied widely, from 0.07 % in Vietnam to 5.10 % in Swaziland. The combined prevalence across all countries categorized in the upper or middle-upper economic strata per the World Bank was 1.00 % (15 countries); the combined lifetime prevalence of countries in lower or lower-middle economic strata (37 countries) was 1.38 %. The total lifetime prevalence of schizophrenia reported across all 52 countries, from a sample size of over 256,000 people, was 1.11 %.

### Point prevalence

Fourteen studies reported the point prevalence of schizophrenia (Additional file 1: Table S3). The median estimate of point prevalence across these studies was 0.32 % (IQR: 0.18 %–0.41 %). The minimum and maximum point prevalence estimates were both among isolated island populations [33, 52].

### Period prevalence other than 12 months or lifetime

Thirteen studies reported prevalence for periods other than 12 months or lifetime (Additional file 1: Table S3). The periods represented ranged from one month to 19 years, with seven studies representing periods greater than one year and six representing periods less than one year (including two with the period not reported). As expected, the median estimate for periods greater than one year (0.39 %; IQR: 0.26 %–0.57 %) fell between those for 12-month and lifetime prevalence, and was higher than the median for periods less than one year (0.20 %; IQR: 0.18 %–0.28 %).

### Within-study estimates

Some trends, such as differences in prevalence methods and case identification, or changes over time, may be better understood by examining differences across estimates within the same study.

#### Prevalence periods and methods

Eight studies compared lifetime estimates to point prevalence or short period prevalence (i.e., ≤12 months) [17, 20, 29, 31, 42, 61, 71, 73]. When the prevalence window was expanded from point or 12 months to an individual’s lifetime, the relative increase in prevalence ranged broadly across studies, from 0 % to 271 % (excluding one study where the six-month prevalence was 0 compared to a 0.6 % lifetime prevalence). Of those seven studies with calculable increases in prevalence, six (85.7 %) reported increases greater than 33 %, and three (42.3 %) observed the prevalence at least double in value. Studies also differed on whether they reported crude or adjusted prevalence, and many reported both crude and adjusted estimates. Among nine studies that reported both crude and age-adjusted prevalence, the age-adjusted estimates were always higher, with relative differences ranging from 17 % to 138 % [19, 21, 39, 40, 47, 55, 72, 77, 78]. The median change was +43 %, and the increase was greater than 66 % for all three lifetime prevalence estimates.

#### Case identification

The use of a broader definition of “schizophrenia spectrum disorders” (including schizophreniform and schizoaffective disorders, versus narrowly defined schizophrenia) increased case identification by 18 %–90 % among six studies, with four studies having increases of 70 % or more [16, 19, 24, 37, 40, 68, 69]. Although studies that only included inpatients were excluded, two Canadian studies compared case identification algorithms that required hospitalizations for schizophrenia to those that included any physician visits; in both studies, inpatient-only lifetime prevalence was approximately 60 % lower than the overall treated lifetime prevalence [71, 74]. The diagnostic classification systems used to identify schizophrenia have evolved over time. Three studies compared multiple classification systems applied to the same populations. In a Swedish study, Lindstrom et al. observed little difference across estimates of schizophrenia prevalence defined by DSM versions III (0.40 %), III-R (0.42 %), and IV (0.43 %), and found that International Classification of Disease, 10^th^ Revision (ICD-10) criteria were slightly more inclusive (0.47 %) than DSM criteria [49]. Similarly, McCreadie et al. reported a higher prevalence in the UK for International Classification of Disease, 9^th^ Revision (ICD-9) schizophrenia (0.33 %) than ICD-10 (0.30 %), which was higher than DSM-III-R prevalence (0.26 %) [50]. Barrett et al., however, found that applying ICD-10 criteria to their Malaysian sample ident`draw 1ified fewer patients than DSM-IV criteria, and that Research Diagnostic Criteria for schizophrenia was the most inclusive [19].

#### Temporal trends

Only one study reported trends in schizophrenia prevalence over time using any data from within the last 15 years. Frisher et al. utilized the General Practice Research Database and found that the annual prevalence of schizophrenia in the UK decreased from 1996 to 2005, from 99.7 per 100,000 patient-years of exposure (PYE) to 74.7 per 100,000 PYE (Chi^2^ for linear trend = 25.7, p < 0.0001) [34]. Two other studies, from Japan [54] and Canada, [74] reported time trends starting in the mid-1980s and spanning a decade, and both suggested an increased schizophrenia prevalence until a peak at the beginning of the 1990s followed by decline in the mid-1990s.

## Discussion

To our knowledge, this study is the first systematic review of the prevalence of schizophrenia among general populations published since 2005. Overall, the median 12-month prevalence of schizophrenia was 0.33 % (IQR: 0.26 %–0.51 %), while the median lifetime prevalence was 0.48 % (IQR: 0.34 %–0.85 %). Estimates showed important variations with different study design, geographic region, study quality, study dates, and other factors, including case identification methods. Some extreme outliers were observed, especially in studies from countries other than Europe and North America; these may reflect true differences in the prevalence of schizophrenia across some populations, potentially due to genetics, geography, socioeconomic differences, different perceptions and levels of awareness, or other factors. This might also be an explanation for the very high variation of schizophrenia prevalence estimates across 52 countries in the WHS. Prevalence estimates were higher for studies with low quality scores, which may indicate that the true prevalence of schizophrenia is lower than estimates reported in lower quality studies. Cohort studies yielded higher prevalence estimates compared to cross-sectional studies. Associations between sample size and prevalence were not observed in the present study, presumably as low sample sizes were excluded and only population-based studies were included. However, the sample size of a study and screening procedures would greatly contribute to the likelihood of identifying cases in a catchment area, particularly with a disease with a relatively low prevalence, such as schizophrenia. Only minor differences in prevalence estimates of schizophrenia calculated using different diagnostic criteria (e.g. ICD-9 vs. ICD-10) were observed in this study. A number of studies showed a 70 % or greater increase, however, when a broader case definition of “schizophrenia spectrum disorders” including schizophreniform and schizoaffective disorders was applied, compared to a narrow case definition of schizophrenia alone. Other studies showed differences by study setting, as inpatient-only lifetime prevalence was approximately 60 % lower than overall (inpatient and outpatient) lifetime prevalence. This evidence suggests that a focus on a sub-group of studies that meet a number of criteria (e.g. study quality and recency, cohort design) may provide a better reflection of the true prevalence of schizophrenia as compared to median or pooled estimates that include older, lower quality studies and apparent outliers such as the prevalence estimates resulting from the WHS.

McGrath highlights challenges with regard to the diagnosis of schizophrenia, with modern diagnostic criteria requiring the exclusion of other general somatic conditions and very varied compliance to screening protocols designed to identify these disorders [1]. These will translate into limitations to separate out measurement error from true variations in prevalence and increasing the variations between estimates [1]. The 2005 review by Saha and colleagues also analyzed factors such as diagnostic criteria, case selection methods, and study quality. It found some differences, but stated that findings were inconclusive [9, 95] Similarly, our findings suggest that design factors contribute to variance in prevalence estimates.

Whereas other reviews may include prevalence estimates from varied populations such as homeless and incarcerated persons, our methods indicate that a thoughtful selection of studies can minimize the variability of some characteristics that typically affect prevalence estimates, improving our understanding of the burden of this disease in the general population. The median lifetime prevalence estimate reported in this review (0.48 %) is similar to, but slightly greater than, the overall prevalence previously reported by Saha and colleagues (0.40 %) [2]. This difference appears to be due to higher estimates among studies published after the search dates of Saha’s review: the median lifetime prevalence among articles published in 2003 or later was 0.51 % (Table 5). Although this study included 28 primary studies from the review by Saha et al., more restrictive selection criteria were applied in this review to compare relatively recent estimates from general populations. Since diagnostic criteria, treatment guidelines, and knowledge about a disease change over time, the restriction to studies published in 1990 or later helped to minimize the impact of these variables on the ascertainment of schizophrenia prevalence. Estimates from this review and the prior review by Saha et al. [2] are less than half the overall estimate reported from the 2003 WHS [56]. As very few studies included in this review reported a prevalence of schizophrenia >1 %, it is possible that the unique study questionnaire used by the WHS, in which respondents self-report previous diagnoses of schizophrenia, led to the differences seen here. Another possible explanation for the higher prevalence estimates reported by the WHS is its use of lay interviewers, who may classify disease differently than psychiatrists, even after the use of standardized reporting forms [96, 97].

**Table 5:** Summary of lifetime prevalence estimates

Strata	N	Min	IQ25	Median	IQ75	Max
Overall	30	0.06 %	0.34 %	0.48 %	0.85 %	5.00 %
Design						
Cross-sectional	18	0.06 %	0.28 %	0.44 %	0.85 %	5.00 %
Cohort	12	0.21 %	0.35 %	0.56 %	0.87 %	1.54 %
Region						
Africa	5	0.06 %	0.40 %	0.47 %	0.80 %	5.00 %
North America	3	0.15 %	0.20 %	0.25 %	0.86 %	1.46 %
Asia	6	0.10 %	0.16 %	0.33 %	0.49 %	0.88 %
Europe	13	0.21 %	0.39 %	0.52 %	0.87 %	1.54 %
Oceania	2	0.40 %	0.49 %	0.58 %	0.66 %	0.75 %
Sample Size						
<5000	5	0.35 %	0.49 %	0.60 %	0.75 %	5.00 %
5000–9999	8	0.10 %	0.15 %	0.40 %	0.61 %	1.30 %
10,000–49,999	7	0.06 %	0.40 %	0.61 %	0.84 %	1.54 %
50,000–99,999	3	0.24 %	0.36 %	0.47 %	0.50 %	0.52 %
100,000+	7	0.21 %	0.29 %	0.41 %	1.16 %	1.46 %
Publication Date						
1990–2002	16	0.06 %	0.24 %	0.40 %	0.66 %	5.00 %
2003–2013	14	0.10 %	0.40 %	0.51 %	0.88 %	1.54 %
Assessment Year^a^						
<1990	10	0.06 %	0.26 %	0.44 %	0.61 %	1.30 %
1990–1999	11	0.15 %	0.32 %	0.40 %	0.61 %	5.00 %
2000–2009	8	0.10 %	0.49 %	0.70 %	0.94 %	1.46 %
Quality Score						
0–7	7	0.13 %	0.23 %	0.75 %	1.00 %	1.30 %
8–10	10	0.06 %	0.35 %	0.45 %	0.59 %	1.54 %
11–15	13	0.10 %	0.39 %	0.47 %	0.80 %	5.00 %

One study (Nuevo et al.) reported on multiple regions, and has been excluded from the “Region” analyses

^a^Selected estimates are the most recent year available. When assessment spanned multiple years, the median year was considered. 4 estimates did not have assessment years reported

In the 2003 WHS, the five lowest prevalence estimates (ranging from 0.07 %–0.27 %) were from Asia and Europe, while the five highest prevalence estimates (ranging from 2.72 %–5.70 %) were all from Africa. It is possible that this reflects differences in the awareness of the disease and case ascertainment methods used across various regions. Interestingly, four of the five other studies in this review that reported lifetime prevalence greater than 1 % were from Canada [71] or Finland [16, 38, 48] (the fifth study was from South Africa), [62] which supports evidence that schizophrenia prevalence may be higher in geographic areas at higher latitudes [98–101].

The choice of study design does play an important role in identifying those in the general community who have not yet been diagnosed with a mental health disorder such as schizophrenia. Birth cohorts, as well as cross-sectional surveys in which mental health professionals interview community members for symptoms indicative of schizophrenia, are time-consuming and expensive to conduct. Alternatively, surveys in which respondents self-report diagnoses are relatively inexpensive, but this method may introduce bias and miss a clinically significant number of undiagnosed cases. Furthermore, the age range of the study samples included in this review varied greatly, which limited our ability to use age range as a variable for sub-analyses. However, since clinicians have realized that schizophrenia symptoms may onset after 45 years of age, studies that restrict the age range of patients potentially underestimate the prevalence of schizophrenia observed in that population.

There is no consensus about how best to summarize observational studies, with relatively little discussion on the strengths and weakness of different approaches [9]. Published systematic reviews on prevalence typically choose different approaches without discussing the rationales of using one method over another [102–106]. We opted to present median values in this study rather than performing a meta-analysis to generate pooled values, as Saha et al stated, “the decision to combine data from randomized controlled trials or risk factor epidemiological studies are of less relevance to prevalence estimates, where estimates based on very large populations should not necessarily carry more weight than estimates based on small populations” [9]. Thus, the variation inherent in the prevalence estimates that have been extracted becomes lost when pooling across studies conducted with different methods, populations, and other variables. Moreover, we performed sub-group analyses instead of a meta-regression analysis as we wanted to compare the difference of prevalence estimates between sub-groups, rather than the size of the effect of factors on the prevalence estimates.

### Limitations

The scope of this review was restricted only to general populations, rather than including focused populations such as patients who have been institutionalized or incarcerated, homeless persons, and migrants. Special populations such as these do have a higher reported prevalence of schizophrenia, but they should be described separately, so as not to overestimate the prevalence in the general population. However, such populations should certainly also be considered by policy makers and healthcare providers to understand the full burden of this disease. Other factors such as socioeconomic status and cannabis use are reported inconsistently in the published literature [69, 70, 95, 107] and were not assessed in the current study, but these and other unmeasured variables may also be associated with the epidemiology of schizophrenia. Another limitation is the exclusion of non-English literature. However, cross-checking English abstracts of excluded studies showed that few studies (including no major studies) were missed given the language restriction which appears to reflect that studies today are commonly published in English.

A number of data gaps became evident in the course of conducting this review. Accurate estimations necessitate the study of sufficiently large populations given the relatively low number of prevalence cases. Several large, heavily populated countries (such as, Brazil, France, Germany, Japan, and Russia) had either one or no published studies on the prevalence of schizophrenia among general populations, while estimates from many other countries were >10 years old and in need of updating. In fact, the only schizophrenia prevalence estimates from Central or South America were the country-specific estimates presented in the 2003 WHS study. The most accurate way to assess schizophrenia prevalence would involve full clinician interviews with the entirety of a population. However, since that is not a feasible method for large populations, a more cost-effective approach could involve screening patients within a nationally-representative survey or registry, and then conducting clinical interviews/examinations to confirm cases; similar methods were employed by Perala and colleagues in Finland [58].

## Conclusions

This updated review provides important evidence on the epidemiology of schizophrenia in general populations, which is vital information for healthcare planning. These data indicate that approximately one in 200 individuals will be diagnosed with schizophrenia at some point during their lifetime. Prevalence estimates across studies varied when looking at different study design, geographic region, time of assessment, and quality scores. As investigator-dependent factors likely lead to variations in published estimates, the present review used a thoughtful selection process of estimates for comparative purposes as well as looking at differences between sub-groups.

Although the size of these variations suggest that study characteristics can influence prevalence estimates, this does not preclude the potential influence of other factors which were not assessed in this study, such as environmental factors. These findings also suggest that a focus on studies that meet a number of criteria (e.g., study quality, recency, and cohort design) may provide a better reflection of the true prevalence of schizophrenia as compared to pooled estimates across very heterogenous studies.

Finally, there is a scarcity of data from many countries, and additional well-designed epidemiological studies performed in these locations will help to improve our understanding of the global prevalence of this disease.

## Additional file

Additional file 1: **Table S1.** Regional Classifications of Countries with Prevalence Estimates. **Table S2.** Evidence Classification. **Table S3.** All Estimates. (DOCX 49 kb)

## Abbreviations

DSM: Diagnostic and Statistical Manual of Mental Disorders
ICD-10: International Classification of Disease, 10^th^ Revision
ICD-9: International Classification of Disease, 9^th^ Revision
IQR: Interquartile range
ISO: International Organization for Standardization
PYE: Patient-years of exposure
UK: United Kingdom
WHS: World Health Survey

## References

[1] McGrath J, Saha S, Chant D, Welham J (2008). Schizophrenia: a concise overview of incidence, prevalence, and mortality. Epidemiol Rev.

[2] Saha S, Chant D, Welham J, McGrath J (2005). A systematic review of the prevalence of schizophrenia. PLoS Med.

[3] Eaton WW (1985). Epidemiology of schizophrenia. Epidemiol Rev.

[4] Eaton WW (1991). Update on the epidemiology of schizophrenia. Epidemiol Rev.

[5] Eaton WW, Tien AY, Poeschla BD, Den Boer JA, Westenberg HGM, van Praag HM (1995). Epidemiology of schizophrenia. Advances in the neurobiology of schizophrenia.

[6] Goldner EM, Hsu L, Waraich P, Somers JM (2002). Prevalence and incidence studies of schizophrenic disorders: a systematic review of the literature. Can J Psychiatry.

[7] Muggah E, Graves E, Bennett C, Manuel DG (2013). Ascertainment of chronic diseases using population health data: a comparison of health administrative data and patient self-report. BMC Public Health.

[8] Vuylsteek K, Hallen M (1994). Epidemiology.

[9] Saha S, Chant D, McGrath J (2008). Meta-analyses of the incidence and prevalence of schizophrenia: conceptual and methodological issues. Int J Methods Psychiatr Res.

[10] Bhati MT (2013). Defining psychosis: the evolution of DSM-5 schizophrenia spectrum disorders. Curr Psychiatry Rep.

[11] Johannessen JO (2003). Review: lifetime prevalence of schizophrenia and related disorders is about 5.5 per 1000, but there is significant variation between regions. Evid Based Ment Health.

[12] Cook DA, West CP (2012). Conducting systematic reviews in medical education: a stepwise approach. Med Educ.

[13] Cook DJ, Mulrow CD, Haynes RB (1997). Systematic reviews: synthesis of best evidence for clinical decisions. Ann Intern Med.

[14] Alessi-Severini S, Biscontri RG, Collins DM, Kozyrskyj A, Sareen J, Enns MW (2008). Utilization and costs of antipsychotic agents: a Canadian population-based study, 1996-2006. Psychiatr Serv.

[15] Al-Uzri MM, Reveley MA, Owen L, Bruce J, Frost S, Mackintosh D (2006). Measuring memory impairment in community-based patients with schizophrenia. Case-control study. Br J Psychiatry.

[16] Arajarvi R, Suvisaari J, Suokas J, Schreck M, Haukka J, Hintikka J (2005). Prevalence and diagnosis of schizophrenia based on register, case record and interview data in an isolated Finnish birth cohort born 1940-1969. Soc Psychiatry Psychiatr Epidemiol.

[17] Awas M, Kebede D, Alem A (1999). Major mental disorders in Butajira, southern Ethiopia. Acta Psychiatr Scand Suppl.

[18] Bamrah JS, Freeman HL, Goldberg DP (1991). Epidemiology of schizophrenia in Salford, 1974-84. Changes in an urban community over ten years. Br J Psychiatry.

[19] Barrett R, Loa P, Jerah E, Nancarrow D, Chant D, Mowry BJ (2005). Rates of treated schizophrenia and its clinical and cultural features in the population isolate of the Iban of Sarawak: A tri-diagnostic approach. Psychol Med.

[20] Bijl RV, Ravelli A, van Zessen G (1998). Prevalence of psychiatric disorder in the general population: results of The Netherlands Mental Health Survey and Incidence Study (NEMESIS). Soc Psychiatry Psychiatr Epidemiol.

[21] Bondestam S, Garssen J, Abdulwakil AI (1990). Prevalence and treatment of mental disorders and epilepsy in Zanzibar. Acta Psychiatr Scand.

[22] Bresee LC, Majumdar SR, Patten SB, Johnson JA (2010). Prevalence of cardiovascular risk factors and disease in people with schizophrenia: a population-based study. Schizophr Res.

[23] Bresee LC, Majumdar SR, Patten SB, Johnson JA (2011). Diabetes, cardiovascular disease, and health care use in people with and without schizophrenia. Eur Psychiatry.

[24] Brown AS, Begg MD, Gravenstein S, Schaefer CA, Wyatt RJ, Bresnahan M (2004). Serologic evidence of prenatal influenza in the etiology of schizophrenia. Arch Gen Psychiatry.

[25] Chang SM, Cho SJ, Jeon HJ, Hahm BJ, Lee HJ, Park JI (2008). Economic burden of schizophrenia in South Korea. J Korean Med Sci.

[26] Chen CN, Wong J, Lee N, Chan-Ho MW, Lau JT, Fung M (1993). The Shatin community mental health survey in Hong Kong. II. Major findings. Arch Gen Psychiatry.

[27] Chien IC, Chou YJ, Lin CH, Bih SH, Chou P, Chang HJ (2004). Prevalence and incidence of schizophrenia among national health insurance enrollees in Taiwan, 1996-2001. Psychiatry Clin Neurosci.

[28] Chien IC, Hsu JH, Lin CH, Bih SH, Chou YJ, Chou P (2009). Prevalence of diabetes in patients with schizophrenia in Taiwan: a population-based National Health Insurance study. Schizophr Res.

[29] Cho MJ, Chang SM, Lee YM, Bae A, Ahn JH, Son J (2010). Prevalence of DSM-IV major mental disorders among Korean adults: a 2006 National Epidemiologic Survey (KECA-R). Asian J Psychiatry.

[30] Cutajar MC, Mullen PE, Ogloff JR, Thomas SD, Wells DL, Spataro J (2010). Schizophrenia and other psychotic disorders in a cohort of sexually abused children. Arch Gen Psychiatry.

[31] de Salvia D, Barbato A, Salvo P, Zadro F (1993). Prevalence and incidence of schizophrenic disorders in Portogruaro. An Italian case register study. J Nerv Ment Dis.

[32] Desai PR, Lawson KA, Barner JC, Rascati KL. Estimating the direct and indirect costs for community-dwelling patients with schizophrenia. J Pharm Health Serv Res. 2013;4(4):187–194.

[33] Fekadu A, Shibre T, Alem A, Kebede D, Kebreab S, Negash A (2004). Bipolar disorder among an isolated island community in Ethiopia. J Affect Disord.

[34] Frisher M, Crome I, Martino O, Croft P (2009). Assessing the impact of cannabis use on trends in diagnosed schizophrenia in the United Kingdom from 1996 to 2005. Schizophr Res.

[35] Goldacre M, Shiwach R, Yeates D (1994). Estimating incidence and prevalence of treated psychiatric disorders from routine statistics: the example of schizophrenia in Oxfordshire. J Epidemiol Community Health.

[36] Goldner EM, Jones W, Waraich P (2003). Using administrative data to analyze the prevalence and distribution of schizophrenic disorders. Psychiatr Serv.

[37] Harvey CA, Pantelis C, Taylor J, McCabe PJ, Lefevre K, Campbell PG (1996). The Camden schizophrenia surveys. II. High prevalence of schizophrenia in an inner London borough and its relationship to socio-demographic factors. Br J Psychiatry.

[38] Hovatta I, Terwilliger JD, Lichtermann D, Makikyro T, Suvisaari J, Peltonen L (1997). Schizophrenia in the genetic isolate of Finland. Am J Med Genet.

[39] Jay M, Gorwood P, Feingold J, Leboyer M (1997). A one year prevalence study of schizophrenia on Reunion Island. Eur Psychiatry.

[40] Jeffreys SE, Harvey CA, McNaught AS, Quayle AS, King MB, Bird AS (1997). The Hampstead Schizophrenia Survey 1991. I: Prevalence and service use comparisons in an inner London health authority, 1986-1991. Br J Psychiatry.

[41] Kake TR, Arnold R, Ellis P (2008). Estimating the prevalence of schizophrenia among New Zealand Maori: a capture-recapture approach. Aust N Z J Psychiatry.

[42] Kebede D, Alem A (1999). Major mental disorders in Addis Ababa, Ethiopia. I. Schizophrenia, schizoaffective and cognitive disorders. Acta Psychiatr Scand Suppl.

[43] Kebede D, Alem A, Shibre T, Negash A, Fekadu A, Fekadu D (2003). Onset and clinical course of schizophrenia in Butajira-Ethiopia--a community-based study. Soc Psychiatry Psychiatr Epidemiol.

[44] Kendler KS, Gallagher TJ, Abelson JM, Kessler RC (1996). Lifetime prevalence, demographic risk factors, and diagnostic validity of nonaffective psychosis as assessed in a US community sample. The National Comorbidity Survey. Arch Gen Psychiatry.

[45] Kendler KS, McGuire M, Gruenberg AM, Walsh D (1994). An epidemiologic, clinical, and family study of simple schizophrenia in County Roscommon, Ireland. Am J Psychiatry.

[46] Kodesh A, Goldshtein I, Gelkopf M, Goren I, Chodick G, Shalev V (2012). Epidemiology and comorbidity of severe mental illnesses in the community: findings from a computerized mental health registry in a large Israeli health organization. Soc Psychiatry Psychiatr Epidemiol.

[47] Kurihara T, Kato M, Reverger R, Tirta IGR, Kashima H (2005). Never-treated patients with schizophrenia in the developing country of Bali. Schizophr Res.

[48] Lehtinen V, Joukamaa M, Lahtela K, Raitasalo R, Jyrkinen E, Maatela J (1990). Prevalence of mental disorders among adults in Finland: basic results from the Mini Finland Health Survey. Acta Psychiatr Scand.

[49] Lindström E, Widerlöv B, von Knorring L (1997). The ICD-10 and DSM-IV diagnostic criteria and the prevalence of schizophrenia. Eur Psychiatry.

[50] McCreadie RG, Leese M, Tilak-Singh D, Loftus L, MacEwan T, Thornicroft G (1997). Nithsdale, Nunhead and Norwood: similarities and differences in prevalence of schizophrenia and utilisation of services in rural and urban areas. Br J Psychiatry.

[51] Moreno B, Garcia-Alonso CR, Negin Hernandez MA, Torres-Gonzalez F, Salvador-Carulla L (2008). Spatial analysis to identify hotspots of prevalence of schizophrenia. Soc Psychiatry Psychiatr Epidemiol.

[52] Myles-Worsley M, Coon H, Tiobech J, Collier J, Dale P, Wender P (1999). Genetic epidemiological study of schizophrenia in Palau, Micronesia: prevalence and familiality. Am J Med Genet.

[53] Najim H (2013). Case identification of severe mental illness in maidstone - a semi-rural setting. Psychiatr Danub.

[54] Nakamura Y, Ojima T, Oki I, Tanihara S, Yanagawa H (1997). Estimation of the future numbers of patients with mental disorders in Japan based on the results of National Patient Surveys. J Epidemiol.

[55] Ni Nuallain M, O'Hare A, Walsh D (1990). The prevalence of schizophrenia in three counties in Ireland. Acta Psychiatr Scand.

[56] Nuevo R, Chatterji S, Verdes E, Naidoo N, Arango C, Ayuso-Mateos JL (2012). The continuum of psychotic symptoms in the general population: a cross-national study. Schizophr Bull.

[57] Ochoa S, Haro JM, Torres JV, Pinto-Meza A, Palacin C, Bernal M (2008). What is the relative importance of self reported psychotic symptoms in epidemiological studies? Results from the ESEMeD--Catalonia Study. Schizophr Res.

[58] Perala J, Suvisaari J, Saarni SI, Kuoppasalmi K, Isometsa E, Pirkola S (2007). Lifetime prevalence of psychotic and bipolar I disorders in a general population. Arch Gen Psychiatry.

[59] Phanthunane P, Vos T, Whiteford H, Bertram M, Udomratn P. Schizophrenia in Thailand: Prevalence and burden of disease. Popul Health Metrics. 2010;8(1):24–31. 10.1186/1478-7954-8-24 PMC2936278 20712909

[60] Phillips MR, Yang G, Li S, Li Y (2004). Suicide and the unique prevalence pattern of schizophrenia in mainland China: a retrospective observational study. Lancet.

[61] Ran MS, Xiang MZ, Li SX, Shan YH, Huang MS, Li SG (2003). Prevalence and course of schizophrenia in a Chinese rural area. Aust N Z J Psychiatry.

[62] Rumble S, Swartz L, Parry C, Zwarenstein M (1996). Prevalence of psychiatric morbidity in the adult population of a rural South African village. Psychol Med.

[63] Schrier AC, van de Wetering BJ, Mulder PG, Selten JP (2001). Point prevalence of schizophrenia in immigrant groups in Rotterdam: data from outpatient facilities. Eur Psychiatry.

[64] Scully PJ, Owens JM, Kinsella A, Waddington JL (2004). Schizophrenia, schizoaffective and bipolar disorder within an epidemiologically complete, homogeneous population in rural Ireland: small area variation in rate. Schizophr Res.

[65] Shibre T, Teferra S, Morgan C, Alem A (2010). Exploring the apparent absence of psychosis amongst the Borana pastoralist community of Southern Ethiopia. A mixed method follow-up study. World Psychiatry.

[66] Shivashankar S, Telfer S, Arunagiriraj J, McKinnon M, Jauhar S, Krishnadas R (2013). Has the prevalence, clinical presentation and social functioning of schizophrenia changed over the last 25years? Nithsdale schizophrenia survey revisited. Schizophr Res.

[67] Stefansson JG, Lindal E, Bjornsson JK, Guomundsdottir A (1991). Lifetime prevalence of specific mental disorders among people born in Iceland in 1931. Acta Psychiatr Scand.

[68] Sutterland AL, Dieleman J, Storosum JG, Voordouw BA, Kroon J, Veldhuis J (2013). Annual incidence rate of schizophrenia and schizophrenia spectrum disorders in a longitudinal population-based cohort study. Soc Psychiatry Psychiatr Epidemiol.

[69] Tizon JL, Ferrando J, Artigue J, Parra B, Pares A, Goma M (2009). Neighborhood differences in psychoses: prevalence of psychotic disorders in two socially-differentiated metropolitan areas of Barcelona. Schizophr Res.

[70] van Os J, Pedersen CB, Mortensen PB (2004). Confirmation of synergy between urbanicity and familial liability in the causation of psychosis. Am J Psychiatry.

[71] Vanasse A, Courteau J, Fleury MJ, Gregoire JP, Lesage A, Moisan J (2012). Treatment prevalence and incidence of schizophrenia in Quebec using a population health services perspective: different algorithms, different estimates. Soc Psychiatry Psychiatr Epidemiol.

[72] Waldo MC (1999). Schizophrenia in Kosrae, Micronesia: prevalence, gender ratios, and clinical symptomatology. Schizophr Res.

[73] Wittchen HU, Essau CA, von Zerssen D, Krieg JC, Zaudig M (1992). Lifetime and six-month prevalence of mental disorders in the Munich Follow-Up Study. Eur Arch Psychiatry Clin Neurosci.

[74] Woogh C (2001). Is schizophrenia on the decline in Canada?. Can J Psychiatry.

[75] Wu EQ, Shi L, Birnbaum H, Hudson T, Kessler R (2006). Annual prevalence of diagnosed schizophrenia in the USA: a claims data analysis approach. Psychol Med.

[76] Xiang YT, Ma X, Cai ZJ, Li SR, Xiang YQ, Guo HL (2008). Prevalence and socio-demographic correlates of schizophrenia in Beijing. Chin Schizophr Res.

[77] Youssef HA, Kinsella A, Waddington JL (1991). Evidence for geographical variations in the prevalence of schizophrenia in rural Ireland. Arch Gen Psychiatry.

[78] Youssef HA, Scully PJ, Kinsella A, Waddington JL (1999). Geographical variation in rate of schizophrenia in rural Ireland by place at birth vs place at onset. Schizophr Res.

[79] Alem A, Kebede D, Fekadu A, Shibre T, Fekadu D, Beyero T (2009). Clinical course and outcome of Schizophrenia in a predominantly treatment-naive cohort in rural ethiopia. Schizophr Bull.

[80] Arajarvi R, Haukka J, Varilo T, Suokas J, Juvonen H, Suvisaari J (2004). Clinical phenotype of schizophrenia in a Finnish isolate. Schizophr Res.

[81] Beyero T, Alem A, Kebede D, Shibire T, Desta M, Deyessa N (2004). Mental disorders among the Borana semi-nomadic community in Southern Ethiopia. World Psychiatry.

[82] Chien IC, Chou YJ, Lin CH, Bih SH, Chou P (2004). Prevalence of psychiatric disorders among National Health Insurance enrollees in Taiwan. Psychiatr Serv.

[83] Cho MJ, Kim JK, Jeon HJ, Suh T, Chung IW, Hong JP (2007). Lifetime and 12-month prevalence of DSM-IV psychiatric disorders among Korean adults. J Nerv Ment Dis.

[84] Fors BM, Isacson D, Bingefors K, Widerlov B (2007). Mortality among persons with schizophrenia in Sweden: an epidemiological study. Nord J Psychiatry.

[85] Gorwood P, Leboyer M, Jay M, Payan C, Feingold J (1995). Gender and age at onset in schizophrenia: impact of family history. Am J Psychiatry.

[86] Perala J, Saarni SI, Ostamo A, Pirkola S, Haukka J, Harkanen T (2008). Geographic variation and sociodemographic characteristics of psychotic disorders in Finland. Schizophr Res.

[87] Spataro J, Mullen PE, Burgess PM, Wells DL, Moss SA (2004). Impact of child sexual abuse on mental health: prospective study in males and females. Br J Psychiatry.

[88] Suvisaari J, Pera J, Saarni S, Juvonen H, Tuulio-Henriksson A, Lonnqvist J (2009). The epidemiology and descriptive and predictive validity of DSM-iV delusional disorder and subtypes of schizophrenia. Clin Schizophr Relat Psychoses.

[89] Suvisaari J, Perala J, Saarni SI, Harkanen T, Pirkola S, Joukamaa M (2008). Type 2 diabetes among persons with schizophrenia and other psychotic disorders in a general population survey. Eur Arch Psychiatry Clin Neurosci.

[90] Telfer S, Shivashankar S, Krishnadas R, McCreadie RG, Kirkpatrick B (2011). Tardive dyskinesia and deficit schizophrenia. Acta Psychiatr Scand.

[91] Viertio S, Laitinen A, Perala J, Saarni SI, Koskinen S, Lonnqvist J (2007). Visual impairment in persons with psychotic disorder. Soc Psychiatry Psychiatr Epidemiol.

[92] Waddington JL, Youssef HA (1994). Evidence for a gender-specific decline in the rate of schizophrenia in rural Ireland over a 50-year period. Br J Psychiatry.

[93] West J, Logan RF, Hubbard RB, Card TR (2006). Risk of schizophrenia in people with coeliac disease, ulcerative colitis and Crohn’s disease: a general population-based study. Aliment Pharmacol Ther.

[94] Widerlov B, Lindstrom E, von Knorring L (1997). One-year prevalence of long-term functional psychosis in three different areas of Uppsala. Acta Psychiatr Scand.

[95] McGrath J, Saha S, Welham J, El Saadi O, MacCauley C, Chant D (2004). A systematic review of the incidence of schizophrenia: the distribution of rates and the influence of sex, urbanicity, migrant status and methodology. BMC Med.

[96] Kessler RC, Birnbaum H, Demler O, Falloon IR, Gagnon E, Guyer M (2005). The prevalence and correlates of nonaffective psychosis in the National Comorbidity Survey Replication (NCS-R). Biol Psychiatry.

[97] Bebbington P, Nayani T (1995). The psychosis screening questionnaire. Int J Methods Psychiatr Res.

[98] Dealberto MJ (2013). Are the rates of schizophrenia unusually high in Canada? A comparison of Canadian and international data. Psychiatry Res.

[99] Amato R, Pinelli M, Monticelli A, Miele G, Cocozza S (2010). Schizophrenia and vitamin D related genes could have been subject to latitude-driven adaptation. BMC Evol Biol.

[100] Kinney DK, Teixeira P, Hsu D, Napoleon SC, Crowley DJ, Miller A (2009). Relation of schizophrenia prevalence to latitude, climate, fish consumption, infant mortality, and skin color: a role for prenatal vitamin d deficiency and infections?. Schizophr Bull.

[101] Saha S, Chant DC, Welham JL, McGrath JJ (2006). The incidence and prevalence of schizophrenia varies with latitude. Acta Psychiatr Scand.

[102] Nandi A, Beard JR, Galea S (2009). Epidemiologic heterogeneity of common mood and anxiety disorders over the lifecourse in the general population: a systematic review. BMC Psychiatry.

[103] Clancy MJ, Clarke MC, Connor DJ, Cannon M, Cotter DR (2014). The prevalence of psychosis in epilepsy; a systematic review and meta-analysis. BMC Psychiatry.

[104] Haller H, Cramer H, Lauche R, Gass F, Dobos GJ (2014). The prevalence and burden of subthreshold generalized anxiety disorder: a systematic review. BMC Psychiatry.

[105] Catala-Lopez F, Peiro S, Ridao M, Sanfelix-Gimeno G, Genova-Maleras R, Catala MA (2012). Prevalence of attention deficit hyperactivity disorder among children and adolescents in Spain: a systematic review and meta-analysis of epidemiological studies. BMC Psychiatry.

[106] Salguero JM, Fernandez-Berrocal P, Iruarrizaga I, Cano-Vindel A, Galea S (2011). Major depressive disorder following terrorist attacks: a systematic review of prevalence, course and correlates. BMC Psychiatry.

[107] McLoughlin BC, Pushpa-Rajah JA, Gillies D, Rathbone J, Variend H, Kalakouti E (2014). Cannabis and schizophrenia. Cochrane Database Syst Rev.

