# Supplementary material for: An evaluation of variation in published estimates of schizophrenia prevalence from 1990─2013: a systematic literature review
Source: BMC Psychiatry. 2015 Aug 12;15:193. doi: 10.1186/s12888-015-0578-7 (PMC4533792; doi:10.1186/s12888-015-0578-7)
Supplement: Additional file 1: — Table S1. Regional Classifications of Countries with Prevalence Estimates. Table S2. Evidence Classification. Table S3. All Estimates. (DOCX 49 kb) [file 12888_2015_578_MOESM1_ESM.docx]

**Supplemental Table 1. Regional Classifications of Countries with Prevalence Estimates**

| **Europe** | **Asia** | **North America** | **Africa** | **Oceania** |
| --- | --- | --- | --- | --- |
| Denmark | China | Canada | Ethiopia | Australia |
| Finland | Indonesia | United States | Reunion Island (France) | Micronesia |
| Iceland | Israel |  | South Africa | New Zealand |
| Ireland | Japan |  | Tanzania |  |
| Italy | Malaysia |  |  |  |
| Netherlands | South Korea |  |  |  |
| Spain | Taiwan |  |  |  |
| Sweden | Thailand |  |  |  |
| United Kingdom |  |  |  |  |
| West Germany [former] |  |  |  |  |

Note: Countries that were only represented in a multinational study (Nuevo et al.) have been excluded

**Supplemental Table 2. Evidence Classification**

| **Author** | **Rate type** | **Case ascertainment** | **Diagnosis** | **Method of diagnostic assignment** | **Information on rates** | **Additional "merits"** | **Total Score** |
| --- | --- | --- | --- | --- | --- | --- | --- |
|  | Rate type mentioned = 1 Rate type not mentioned = 0 | Community survey or multiple institutions = 2 Hospital inpatient & outpatients, case registers = 1 Not specified = 0 | Any diagnostic system reported (e.g., CATEGO, DSM, Feighner, RDC, ICD, local guidelines) = 1 Own system/symptoms described = 0 No system/not specified = 0 | Diagnostic interview (face to face) = 3 Case note review (standardized) = 2 Clinical diagnosis (recorded in hospital notes or registries) = 1 Unspecified = 0 | Raw data-numerator =+1 Raw data-denominator =+1 Age and/or gender standardized =+1 If age/gender standardized, method provided =+1 Confidence intervals =+1 Numerator/denominator match in time =+1 Numerator/denominator match in space =+1 | Text on inter-rater reliability = +1 |  |
| Cutajar, 2010 | 1 | 1 | 1 | 1 | 3 | 0 | **7** |
| Alessi-Severini, 2008 | 1 | 1 | 1 | 1 | 0 | 0 | **4** |
| Bresee, 2010 | 1 | 1 | 1 | 1 | 4 | 0 | **9** |
| Bresee, 2011 | 1 | 2 | 0 | 3 | 4 | 0 | **10** |
| Goldner, 2003 | 1 | 1 | 1 | 1 | 3 | 0 | **7** |
| Vanasse, 2012 | 1 | 1 | 1 | 1 | 5 | 0 | **9** |
| Woogh, 2001 | 1 | 2 | 1 | 1 | 1 | 0 | **6** |
| Chen, 1993 | 1 | 2 | 1 | 3 | 0 | 0 | **7** |
| Phillips, 2004 | 1 | 2 | 1 | 3 | 6 | 0 | **13** |
| Ran, 2003 | 1 | 2 | 1 | 3 | 4 | 1 | **12** |
| Xiang, 2008 | 1 | 2 | 1 | 3 | 6 | 1 | **14** |
| van Os, 2004 | 1 | 1 | 1 | 1 | 4 | 0 | **8** |
| Awas, 1999 | 1 | 2 | 1 | 3 | 4 | 0 | **11** |
| Fekadu, 2004 | 0 | 2 | 1 | 3 | 4 | 1 | **11** |
| Kebede, 1999 | 1 | 2 | 1 | 3 | 4 | 0 | **11** |
| Kebede, 2003 | 1 | 2 | 1 | 3 | 4 | 0 | **11** |
| Shibre, 2010 | 1 | 2 | 1 | 3 | 1 | 0 | **8** |
| Arajarvi, 2005 | 1 | 2 | 1 | 2 | 4 | 0 | **10** |
| Hovatta, 1997 | 1 | 2 | 1 | 2 | 6 | 0 | **12** |
| Lehtinen, 1990 | 0 | 2 | 1 | 3 | 1 | 0 | **7** |
| Perala, 2007 | 1 | 2 | 1 | 3 | 7 | 0 | **14** |
| Wittchen, 1992 | 1 | 2 | 1 | 3 | 4 | 1 | **12** |
| Stefansson, 1991 | 1 | 2 | 1 | 3 | 4 | 0 | **11** |
| Kurihara, 2005 | 1 | 2 | 1 | 3 | 4 | 0 | **11** |
| Kendler, 1994 | 1 | 1 | 1 | 1 | 4 | 1 | **9** |
| Ni Nuallain, 1990 | 1 | 1 | 1 | 3 | 5 | 0 | **11** |
| Scully, 2004 | 1 | 2 | 1 | 3 | 4 | 0 | **11** |
| Youssef, 1991 | 1 | 1 | 1 | 3 | 5 | 0 | **11** |
| Youssef, 1999 | 1 | 1 | 1 | 3 | 6 | 0 | **12** |
| Kodesh, 2012 | 1 | 1 | 1 | 1 | 3 | 0 | **7** |
| de Salvia, 1993 | 1 | 1 | 1 | 1 | 4 | 0 | **8** |
| Nakamura, 1997 | 0 | 1 | 1 | 1 | 0 | 0 | **3** |
| Barrett, 2005 | 1 | 1 | 1 | 2 | 6 | 0 | **11** |
| Waldo, 1999 | 1 | 1 | 1 | 3 | 4 | 0 | **10** |
| Kake, 2008 | 1 | 1 | 1 | 1 | 4 | 0 | **8** |
| Myles-Worsley, 1999 | 1 | 1 | 1 | 3 | 4 | 0 | **10** |
| Jay, 1997 | 1 | 2 | 1 | 2 | 3 | 0 | **9** |
| Rumble, 1996 | 1 | 2 | 1 | 3 | 4 | 1 | **12** |
| Chang, 2008 | 1 | 1 | 1 | 1 | 3 | 0 | **7** |
| Cho, 2010 | 1 | 1 | 1 | 3 | 5 | 0 | **11** |
| Moreno, 2008 | 1 | 1 | 1 | 1 | 4 | 0 | **8** |
| Ochoa, 2008 | 1 | 2 | 1 | 1 | 5 | 0 | **10** |
| Tizon, 2009 | 1 | 2 | 1 | 1 | 5 | 0 | **10** |
| Lindström, 1997 | 1 | 1 | 1 | 2 | 4 | 0 | **9** |
| Chien, 2004 | 1 | 1 | 1 | 1 | 4 | 0 | **8** |
| Chien, 2009 | 0 | 1 | 1 | 1 | 4 | 0 | **7** |
| Bondestam, 1990 | 1 | 2 | 0 | 3 | 2 | 0 | **8** |
| Phanthunane, 2010 | 1 | 2 | 0 | 0 | 1 | 0 | **4** |
| Bijl, 1998 | 1 | 2 | 1 | 3 | 2 | 0 | **9** |
| Schrier, 2001 | 1 | 1 | 1 | 1 | 3 | 0 | **7** |
| Sutterland, 2013 | 1 | 1 | 1 | 1 | 3 | 0 | **7** |
| Al-Uzri, 2006 | 0 | 2 | 1 | 1 | 3 | 0 | **7** |
| Bamrah, 1991 | 1 | 2 | 1 | 2 | 4 | 0 | **10** |
| Frisher, 2009 | 1 | 1 | 1 | 1 | 0 | 0 | **4** |
| Goldacre, 1994 | 1 | 1 | 1 | 1 | 1 | 0 | **5** |
| Harvey, 1996 | 1 | 2 | 1 | 0 | 1 | 0 | **5** |
| Jeffreys, 1997 | 1 | 2 | 1 | 3 | 6 | 0 | **13** |
| McCreadie, 1997 | 0 | 2 | 1 | 2 | 1 | 0 | **6** |
| Najim, 2013 | 0 | 2 | 1 | 1 | 1 | 0 | **5** |
| Shivashankar, 2013 | 1 | 1 | 1 | 3 | 3 | 0 | **9** |
| Brown, 2004 | 1 | 1 | 1 | 3 | 4 | 0 | **10** |
| Desai, 2013 | 1 | 2 | 1 | 1 | 3 | 0 | **8** |
| Kendler, 1996 | 1 | 2 | 1 | 3 | 1 | 1 | **9** |
| Wu, 2006 | 1 | 1 | 1 | 1 | 1 | 0 | **5** |
| Nuevo, 2012 | 1 | 2 | 0 | 0 | 4 | 0 | **7** |

**Supplemental Table 3. All Estimates**

| **Author, Year** | **Region** | **Country/ Region** | **Schizophrenia Definition** | **Year(s) of Prevalence Assessment** | **Prevalence Period** | **Selected Estimate?** | **Crude Prevalence (95% CI)** | **Adjusted Prevalence (95% CI)** | **Adjustment Factors** | **Study Design** | **Type of Prevalence** |
| --- | --- | --- | --- | --- | --- | --- | --- | --- | --- | --- | --- |
| Cutajar, 2010 | Oceania | Victoria, Australia | Schizophrenia, schizoaffective disorder, or delusional disorders | NR | Lifetime | Yes | 0.75% | NR | NR | Cohort | Treated/ diagnosed |
| Alessi-Severini, 2008 | North America | Manitoba, Canada | ICD-9 295 | 1996–2006 | 12 months | Yes | 0.6% | NR | NR | Cohort | Treated/ diagnosed |
| Bresee, 2010 | North America | Alberta, Canada | ICD-9 295, ICD-10 F20 | 1995–2006 | 12 years | Yes | 1.24% | NR | NR | Cohort | Treated/ diagnosed |
| Bresee, 2011 | North America | Canada | Schizophrenia, self-report | 2005 | Point | Yes | 0.33% | NR | NR | Cross-sectional | Treated/ diagnosed |
| Goldner, 2003 | North America | British Columbia, Canada | ICD-9 295, DSM-IV schizophrenia | 1996–1997 | 12 months |  | 0.45% | NR | NR | Cohort | Treated/ diagnosed |
| Goldner, 2003 | North America | British Columbia, Canada | ICD-9 295, DSM-IV schizophrenia | 1997–1998 | 12 months |  | 0.45% | NR | NR | Cohort | Treated/ diagnosed |
| Goldner, 2003 | North America | British Columbia, Canada | ICD-9 295, DSM-IV schizophrenia | 1998–1999 | 12 months | Yes | 0.42% | NR | NR | Cohort | Treated/ diagnosed |
| Goldner, 2003 | North America | Vancouver, Canada | ICD-9 295, DSM-IV schizophrenia | 1996–1997 | 12 months |  | 0.64% | NR | NR | Cohort | Treated/ diagnosed |
| Goldner, 2003 | North America | Vancouver, Canada | ICD-9 295, DSM-IV schizophrenia | 1997–1998 | 12 months |  | 0.61% | NR | NR | Cohort | Treated/ diagnosed |
| Goldner, 2003 | North America | Vancouver, Canada | ICD-9 295, DSM-IV schizophrenia | 1998–1999 | 12 months |  | 0.60% | NR | NR | Cohort | Treated/ diagnosed |
| Vanasse, 2012 | North America | Quebec, Canada | Hospitalization with ICD-9: 295; ICD-10: F20, F21, F23.2, F25 | 2006 | 12 months |  | 0.13% (0.13%–0.14%) | NR | NR | Cohort | Treated/ diagnosed |
| Vanasse, 2012 | North America | Quebec, Canada | Hospitalization or ER visit with ICD-9: 295; ICD-10: F20, F21, F23.2, F25 | 2006 | 12 months |  | 0.20% (0.20%–0.20%) | NR | NR | Cohort | Treated/ diagnosed |
| Vanasse, 2012 | North America | Quebec, Canada | Hospitalization or psychiatrist visit with ICD-9: 295; ICD-10: F20, F21, F23.2, F25 | 2006 | 12 months |  | 0.44% (0.44%–0.45%) | NR | NR | Cohort | Treated/ diagnosed |
| Vanasse, 2012 | North America | Quebec, Canada | Hospitalization or any physician visit with ICD-9: 295; ICD-10: F20, F21, F23.2, F25 | 2006 | 12 months | Yes | 0.56% (0.56%–0.57%) | NR | NR | Cohort | Treated/ diagnosed |
| Vanasse, 2012 | North America | Quebec, Canada | Hospitalization with ICD-9: 295; ICD-10: F20, F21, F23.2, F25 | 1996–2006 | Lifetime |  | 0.59% (0.59%–0.60%) | NR | NR | Cohort | Treated/ diagnosed |
| Vanasse, 2012 | North America | Quebec, Canada | Hospitalization or ER visit with ICD-9: 295; ICD-10: F20, F21, F23.2, F25 | 1996–2006 | Lifetime |  | 0.87% (0.86%–0.88%) | NR | NR | Cohort | Treated/ diagnosed |
| Vanasse, 2012 | North America | Quebec, Canada | Hospitalization or psychiatrist visit with ICD-9: 295; ICD-10: F20, F21, F23.2, F25 | 1996–2006 | Lifetime |  | 1.18% (1.17%–1.19%) | NR | NR | Cohort | Treated/ diagnosed |
| Vanasse, 2012 | North America | Quebec, Canada | Hospitalization or any physician visit with ICD-9: 295; ICD-10: F20, F21, F23.2, F25 | 1996–2006 | Lifetime | Yes | 1.46% (1.45%–1.47%) | NR | NR | Cohort | Treated/ diagnosed |
| Woogh, 2001 | North America | Kingston, Canada | DSM-IV schizophrenia | 1986–1987 | Lifetime |  | 0.35% | NR | NR | Cohort | Treated/ diagnosed |
| Woogh, 2001 | North America | Kingston, Canada | DSM-IV schizophrenia | 1991–1992 | Lifetime |  | 0.39% | NR | NR | Cohort | Treated/ diagnosed |
| Woogh, 2001 | North America | Kingston, Canada | DSM-IV schizophrenia | 1996–1997 | Lifetime | Yes | 0.25% | NR | NR | Cohort | Treated/ diagnosed |
| Woogh, 2001 | North America | Kingston, Canada | DSM-IV schizophrenia, inpatients | 1986–1987 | Lifetime |  | 0.21% | NR | NR | Cohort | Treated/ diagnosed |
| Woogh, 2001 | North America | Kingston, Canada | DSM-IV schizophrenia, inpatients | 1991–1992 | Lifetime |  | 0.16% | NR | NR | Cohort | Treated/ diagnosed |
| Woogh, 2001 | North America | Kingston, Canada | DSM-IV schizophrenia, inpatients | 1996–1997 | Lifetime |  | 0.10% | NR | NR | Cohort | Treated/ diagnosed |
| Woogh, 2001 | North America | Kingston, Canada | DSM-IV schizophrenia, outpatients/ER | 1986–1987 | Lifetime |  | 0.14% | NR | NR | Cohort | Treated/ diagnosed |
| Woogh, 2001 | North America | Kingston, Canada | DSM-IV schizophrenia, outpatients/ER | 1991–1992 | Lifetime |  | 0.14% | NR | NR | Cohort | Treated/ diagnosed |
| Woogh, 2001 | North America | Kingston, Canada | DSM-IV schizophrenia, outpatients/ER | 1996–1997 | Lifetime |  | 0.15% | NR | NR | Cohort | Treated/ diagnosed |
| Chen, 1993 | Asia | Shatin (Hong Kong), China | DSM-III schizophrenic disorder, via DIS | 1984–1986 | Lifetime | Yes | NR | 0.12%–0.13% (SE: 0.06) | Selection bias | Cross-sectional | Overall |
| Phillips, 2004 | Asia | China | ICD-9 schizophrenia, via PSE-9 | 1993 | Point | Yes | 0.53% | 0.47% (0.38%–0.59%) | Location, by gender distribution (according to 1995–1999 census) | Cross-sectional | Overall |
| Ran, 2003 | Asia | Rural Xinjin County, China | CCMD-2-R, ICD-10 schizophrenia | 1994 | Point | Yes | 0.30% | NR | NR | Cross-sectional | Overall |
| Ran, 2003 | Asia | Rural Xinjin County, China | CCMD-2-R, ICD-10 schizophrenia | 1994 | Lifetime | Yes | 0.41% | NR | NR | Cross-sectional | Overall |
| Ran, 2003 | Asia | Rural Xinjin County, China | CCMD-2-R, ICD-10 schizophrenia | 1994 | Lifetime |  | 0.29% | NR | NR | Cross-sectional | Treated |
| Ran, 2003 | Asia | Rural Xinjin County, China | CCMD-2-R, ICD-10 schizophrenia | 1994 | Point |  | 0.02% | NR | NR | Cross-sectional | Antipsychotic treated |
| Xiang, 2008 | Asia | Beijing, China | ICD-10 schizophrenia, via CIDI | 2003 | Lifetime | Yes | 0.52% | 0.49% (0.30%–0.68%) | Age, gender | Cross-sectional | Overall |
| Xiang, 2008 | Asia | Beijing, China | ICD-10 schizophrenia, via CIDI | 2003 | Lifetime |  | 0.30% | NR | NR | Cross-sectional | Treated |
| van Os, 2004 | Europe | Denmark | ICD-8 295, ICD-10 F20 | 1975–2001 | Lifetime | Yes | 0.33% | NR | NR | Cohort | Treated/ diagnosed |
| Awas, 1999 | Africa | Butajira, Ethiopia | ICD-10 F20, via CIDI | 1996 | Lifetime | Yes | NR | 0.8% | Selection bias | Cross-sectional | Overall |
| Awas, 1999 | Africa | Butajira, Ethiopia | ICD-10 F20, via CIDI | 1996 | 1 month | Yes | NR | 0.6% | Selection bias | Cross-sectional | Overall |
| Fekadu, 2004 | Africa | Zeway islands, Ethiopia | ICD-10 schizophrenia | 1998 | Point | Yes | 0.06% | NR | NR | Cross-sectional | Overall |
| Kebede, 1999 | Africa | Addis Ababa, Ethiopia | ICD-10 F20, via CIDI | 1994 | Lifetime | Yes | NR | 0.4% | Selection bias | Cross-sectional | Overall |
| Kebede, 1999 | Africa | Addis Ababa, Ethiopia | ICD-10 F20, via CIDI | 1994 | 1 month | Yes | NR | 0.3% | Selection bias | Cross-sectional | Overall |
| Kebede, 1999 | Africa | Addis Ababa, Ethiopia | ICD-10 F20, F25.0, F25.1, or F25.2, via CIDI | 1994 | Lifetime |  | NR | 0.9% | Selection bias | Cross-sectional | Overall |
| Kebede, 2003 | Africa | Addis Ababa, Ethiopia | ICD-10 F20, F25.0, F25.1, or F25.2, via CIDI | 1994 | 1 month |  | NR | 0.7% | Selection bias | Cross-sectional | Overall |
| Kebede, 2003 | Africa | Butajira, Ethiopia | ICD-10 F20, via SCAN | 2001 | Lifetime | Yes | 0.47% | NR | NR | Cross-sectional | Overall |
| Kebede, 2003 | Africa | Butajira, Ethiopia | ICD-10 F20, via SCAN | 2001 | Lifetime |  | 0.05% | NR | NR | Cross-sectional | Treated |
| Shibre, 2010 | Africa | Borana community, Ethiopia | DSM-IV schizophrenia, via SCAN | NR | Point | Yes | 0.08% | NR | NR | Cross-sectional | Overall |
| Arajarvi, 2005 | Europe | Kuusamo, Finland | ICD-8, ICD-9, ICD-10 schizophrenia | 1998 | Lifetime | Yes | 1.54% | 0.91%–1.32% | Consensus diagnosis | Cohort | Treated/ diagnosed |
| Arajarvi, 2005 | Europe | Kuusamo, Finland | ICD-8, ICD-9, ICD-10 schizophrenia spectrum disorders | 1998 | Lifetime |  | 1.92% | 1.15%–1.66% | Consensus diagnosis | Cohort | Treated/ diagnosed |
| Hovatta, 1997 | Europe | Finland | ICD-8, ICD-9 schizophrenia | 1974–1991 | Lifetime | Yes | 1.21% | 1.1% | Age-corrected lifetime risk | Cohort | Treated/ diagnosed |
| Lehtinen, 1990 | Europe | Finland | Definite or probable schizophrenia | 1980 | Lifetime | Yes | NR | 1.3% | Age | Cross-sectional | Overall |
| Perala, 2007 | Europe | Finland | DSM-IV schizophrenia, via SCAN | 2002–2004 | Lifetime | Yes | NR | 0.87% (0.68%–1.11%) | Selection bias, correlation within clusters, Age (correction of elderly oversampling) | Cross-sectional | Overall |
| Wittchen, 1992 | Europe | West Germany [former] | DSM-III schizophrenia, via DIS | 1981 | Lifetime | Yes | NR | 0.60% (SE: 0.29) | Selection bias | Cohort | Overall |
| Wittchen, 1992 | Europe | West Germany [former] | DSM-III schizophrenia, via DIS | 1981 | 6 months | Yes | NR | 0% | NR | Cohort | Overall |
| Wittchen, 1992 | Europe | West Germany [former] | DSM-III schizophrenia/schizophreniform, via DIS | 1981 | Lifetime |  | NR | 0.71% (SE: 0.30) | Selection bias | Cohort | Overall |
| Wittchen, 1992 | Europe | West Germany [former] | DSM-III schizophrenia/schizophreniform, via DIS | 1981 | 6 months |  | NR | 0% | NR | Cohort | Overall |
| Wittchen, 1992 | Europe | West Germany [former] | ICD-9 295.0, 297.0, 298.0 | 1981 | Lifetime |  | NR | 0.71% (SE: 0.30) | Selection bias | Cohort | Overall |
| Stefansson, 1991 | Europe | Iceland | DSM-III schizophrenia or schizophreniform disorders, via DIS | 1987–1988 | Lifetime | Yes | 0.35% (SE: 0.2) | NR | NR | Cohort | Overall |
| Kurihara, 2005 | Asia | Rural Bali, Indonesia | DSM-III-R schizophrenia, via SCID | 2001–2002 | Point | Yes | 0.42% | 0.60% | Age | Cross-sectional | Overall |
| Kurihara, 2005 | Asia | Rural Bali, Indonesia | DSM-III-R schizophrenia or schizophreniform disorder, via SCID | 2001–2002 | Point |  | 0.46% | 0.65% | Age | Cross-sectional | Overall |
| Kurihara, 2005 | Asia | Rural Bali, Indonesia | DSM-III-R schizophrenia or schizophreniform disorder, via SCID | 2001–2002 | Point |  | 0.22% | NR | NR | Cross-sectional | Treated |
| Kendler, 1994 | Europe | Roscommon County, Ireland | DSM-III-R schizophrenia | 1986 | Lifetime | Yes | 0.61% | NR | NR | Cohort | Treated/ diagnosed |
| Ni Nuallain, 1990 | Europe | Carlow/South Kildare, Westmeath, and Roscommon counties, Ireland | PSE S class | 1974–1977 | 12 months |  | 0.08% | 0.10% | Age | Cohort | Treated/ diagnosed |
| Ni Nuallain, 1990 | Europe | Carlow/South Kildare, Westmeath, and Roscommon counties, Ireland | PSE S+P classes | 1974–1977 | 12 months |  | 0.10% | 0.12% | Age | Cohort | Treated/ diagnosed |
| Ni Nuallain, 1990 | Europe | Carlow/South Kildare, Westmeath, and Roscommon counties, Ireland | PSE/SCL S class | 1974–1977 | 12 months | Yes | 0.33% | 0.39% | Age | Cohort | Treated/ diagnosed |
| Ni Nuallain, 1990 | Europe | Carlow/South Kildare, Westmeath, and Roscommon counties, Ireland | PSE/SCL S+P classes | 1974–1977 | 12 months |  | 0.44% | 0.52% | Age | Cohort | Treated/ diagnosed |
| Ni Nuallain, 1990 | Europe | Carlow/South Kildare, Westmeath, and Roscommon counties, Ireland | ICD-8 295, 297, 298.3, 298.4 | 1974 | 12 months |  | 0.62% | 0.73% | Age | Cohort | Treated/ diagnosed |
| Scully, 2004 | Europe | Monaghan, Ireland | DSM-III-R schizophrenia, via SCID | 1996 | Lifetime | Yes | 0.39% (SE: 0.04) | 7.3 per 1,000 risk-lives-exposed (SE: 0.7) | Morbid risk | Cross-sectional | Overall |
| Scully, 2004 | Europe | Monaghan, Ireland | DSM-III-R schizophrenia or schizoaffective disorder, via SCID | 1996 | Lifetime |  | 0.50% | NR | Morbid risk | Cross-sectional | Overall |
| Youssef, 1991 | Europe | County Cavan, Ireland | DSM-III-R schizophrenia | 1987–1988 | 12 months | Yes | 0.33% | 0.46; 0.64 per 100 risk-lives-exposed | Age/morbid risk | Cross-sectional | Treated/ diagnosed |
| Youssef, 1999 | Europe | County Monaghan, Ireland | DSM-III-R schizophrenia (by place at onset) | 1992 | 12 months | Yes | 0.34% (SE: 0.04) | 0.47% (SE: 0.06); 0.66 per 100 risk-lives-exposed (SE: 0.08) | Age/morbid risk | Cross-sectional | Treated/ diagnosed |
| Youssef, 1999 | Europe | County Monaghan, Ireland | DSM-III-R schizophrenia (by place at birth) | 1992 | 12 months |  | 0.37% (SE: 0.05) | 0.58% (SE: 0.07); 0.72 per 100 risk-lives-exposed (SE: 0.09) | Age/morbid risk | Cross-sectional | Treated/ diagnosed |
| Kodesh, 2012 | Asia | Israel | ICD-9 schizophrenia and schizoaffective disorders | 2003–2009 | 7 years | Yes | 0.5% | NR | NR | Cohort | Treated/ diagnosed |
| de Salvia, 1993 | Europe | Portogruaro, Italy | ICD-9 295, 297, 298.3, 298.4, 298.8, 298.9, 299 | 1982–1989 | Point | Yes | 0.14% | NR | NR | Cohort | Treated/ diagnosed |
| de Salvia, 1993 | Europe | Portogruaro, Italy | ICD-9 295, 297, 298.3, 298.4, 298.8, 298.9, 299 | 1982–1989 | 12 months | Yes | 0.27% | NR | NR | Cohort | Treated/ diagnosed |
| de Salvia, 1993 | Europe | Portogruaro, Italy | ICD-9 295, 297, 298.3, 298.4, 298.8, 298.9, 299 | 1989 | Lifetime | Yes | 0.52% | NR | NR | Cohort | Treated/ diagnosed |
| Nakamura, 1997 | Asia | Japan | ICD-9 295 | 1984 | Point |  | 0.34% | NR | NR | Cross-sectional | Treated/ diagnosed |
| Nakamura, 1997 | Asia | Japan | ICD-9 295 | 1987 | Point |  | 0.35% | NR | NR | Cross-sectional | Treated/ diagnosed |
| Nakamura, 1997 | Asia | Japan | ICD-9 295 | 1990 | Point |  | 0.39% | NR | NR | Cross-sectional | Treated/ diagnosed |
| Nakamura, 1997 | Asia | Japan | ICD-9 295 | 1993 | Point | Yes | 0.37% | NR | NR | Cross-sectional | Treated/ diagnosed |
| Barrett, 2005 | Asia | Sarawak, Malaysia | ICD-10 narrow schizophrenia | 1960–2000 | Lifetime |  | 0.18% | 0.42% | Age | Cross-sectional | Treated/ diagnosed |
| Barrett, 2005 | Asia | Sarawak, Malaysia | DSM-IV narrow schizophrenia | 1960–2000 | Lifetime | Yes | 0.24% | 0.57% | Age | Cross-sectional | Treated/ diagnosed |
| Barrett, 2005 | Asia | Sarawak, Malaysia | RDC narrow schizophrenia | 1960–2000 | Lifetime |  | 0.34% | 0.83% | Age | Cross-sectional | Treated/ diagnosed |
| Barrett, 2005 | Asia | Sarawak, Malaysia | ICD-10 schizophrenia spectrum disorders | 1960–2000 | Lifetime |  | 0.42% | 0.42% | Age | Cross-sectional | Treated/ diagnosed |
| Barrett, 2005 | Asia | Sarawak, Malaysia | DSM-IV schizophrenia spectrum disorders | 1960–2000 | Lifetime |  | 0.42% | 0.57% | Age | Cross-sectional | Treated/ diagnosed |
| Barrett, 2005 | Asia | Sarawak, Malaysia | RDC schizophrenia spectrum disorders | 1960–2000 | Lifetime |  | 0.44% | 0.83% | Age | Cross-sectional | Treated/ diagnosed |
| Waldo, 1999 | Oceania | Kosrae, Micronesia | DSM-IV schizophrenia | 1997 | Lifetime | Yes | 0.40% | 0.68% | Age | Cohort | Overall |
| Kake, 2008 | Oceania | New Zealand | ICD-9 295, excluding 295.4 and 295.7 | 2002–2003 | 12 months | Yes | 0.10% | NR | NR | Cohort | Treated/ diagnosed |
| Myles-Worsley, 1999 | Oceania | Palau, Micronesia | RDC strictly defined schizophrenia | NR | Point | Yes | 1.16% | 1.99% | Lifetime morbid risk | Cohort | Overall |
| Jay, 1997 | Africa | Saint-Denis, Reunion Island (France) | DSM-III-R schizophrenia | 1988 | 12 months | Yes | 0.75% | 1.49% (SE: 0.06) | Age | Cross-sectional | Overall |
| Rumble, 1996 | Africa | Mamre, South Africa | ICD-8 paranoid schizophrenia, via PSE-9 | 1992 | Lifetime | Yes | NR | 5% | Selection bias | Cross-sectional | Overall |
| Chang, 2008 | Asia | South Korea | ICD-10 F20 | 2005 | 12 months | Yes | 0.4% | NR | NR | Cohort | Treated/ diagnosed |
| Cho, 2010 | Asia | South Korea | DSM-IV schizophrenia only, via CIDI | 2006–2007 | Lifetime | Yes | NR | 0.1% | Age, gender | Cross-sectional | Overall |
| Cho, 2010 | Asia | South Korea | DSM-IV schizophrenia only, via CIDI | 2006–2007 | 12 months | Yes | NR | 0.1% | Age, gender | Cross-sectional | Overall |
| Moreno, 2008 | Europe | South Granada, Spain | ICD-10 F20–F29 | 1999 | 12 months | Yes | NR | 0.29% | Age | Cohort | Treated/ diagnosed |
| Ochoa, 2008 | Europe | Catalonia, Spain | DSM-IV schizophrenia, via SCID | 2001–2002 | Lifetime | Yes | 0.49% | 0.48% (0.15%–0.82%) | Population demographics, selection bias | Cross-sectional | Overall |
| Tizon, 2009 | Europe | Barcelona, Spain | DSM-IV schizophrenia, schizophreniform, or schizoaffective disorder | 1982–2000 | 19 years |  | 0.46% (0.42%–0.50%) | NR | NR | Cohort | Treated/ diagnosed |
| Tizon, 2009 | Europe | Barcelona, Spain | DSM-IV schizophrenia only | 1982–2000 | 19 years | Yes | 0.39% | NR | NR | Cohort | Treated/ diagnosed |
| Lindström, 1997 | Europe | Uppsala, Sweden | DSM-III-R schizophrenia | 1991 | 12 months |  | 0.42% | NR | NR | Cohort | Treated/ diagnosed |
| Lindström, 1997 | Europe | Uppsala, Sweden | DSM-III schizophrenia | 1991 | 12 months |  | 0.40% | NR | NR | Cohort | Treated/ diagnosed |
| Lindström, 1997 | Europe | Uppsala, Sweden | DSM-IV schizophrenia | 1991 | 12 months | Yes | 0.43% | NR | NR | Cohort | Treated/ diagnosed |
| Lindström, 1997 | Europe | Uppsala, Sweden | ICD-10 schizophrenia | 1991 | 12 months |  | 0.47% | NR | NR | Cohort | Treated/ diagnosed |
| Chien, 2004 | Asia | Taiwan | ICD-9 295 | 1996 | 12 months | Yes | 0.33% | NR | NR | Cohort | Treated/ diagnosed |
| Chien, 2004 | Asia | Taiwan | ICD-9 295 | 1996–1997 | 2 years |  | 0.43% | NR | NR | Cohort | Treated/ diagnosed |
| Chien, 2004 | Asia | Taiwan | ICD-9 295 | 1996–1998 | 3 years |  | 0.49% | NR | NR | Cohort | Treated/ diagnosed |
| Chien, 2004 | Asia | Taiwan | ICD-9 295 | 1996–1999 | 4 years |  | 0.55% | NR | NR | Cohort | Treated/ diagnosed |
| Chien, 2004 | Asia | Taiwan | ICD-9 295 | 1996–2000 | 5 years |  | 0.60% | NR | NR | Cohort | Treated/ diagnosed |
| Chien, 2004 | Asia | Taiwan | ICD-9 295 | 1996–2001 | 6 years | Yes | 0.64% | NR | NR | Cohort | Treated/ diagnosed |
| Chien, 2009 | Asia | Taiwan | ICD-9 295 | 2005 | 12 months | Yes | 0.58% | NR | NR | Cohort | Treated/ diagnosed |
| Bondestam, 1990 | Africa | Zanzibar, Tanzania | Schizophrenia, clinically established diagnosis | 1988 | Lifetime | Yes | 0.06% | 0.10% | Age | Cross-sectional | Overall |
| Phanthunane, 2010 | Asia | Thailand | Schizophrenia, schizoaffective, or schizophreniform disorder; estimated from non-affective psychosis, via MINI | 2003 | Lifetime | Yes | NR | 0.88% (0.72%–1.06%) | Estimated from psychosis cases | Cross-sectional | Overall |
| Bijl, 1998 | Europe | Netherlands | DSM-III-R schizophrenia, via CIDI | 1996 | Lifetime | Yes | NR | 0.4% | Weighted | Cross-sectional | Overall |
| Bijl, 1998 | Europe | Netherlands | DSM-III-R schizophrenia, via CIDI | 1996 | 12 months | Yes | NR | 0.2% | Weighted | Cross-sectional | Overall |
| Bijl, 1998 | Europe | Netherlands | DSM-III-R schizophrenia, via CIDI | 1996 | 1 month | Yes | NR | 0.2% | Weighted | Cross-sectional | Overall |
| Schrier, 2001 | Europe | Rotterdam, Netherlands | DSM-III-R schizophrenia | 1994 | Lifetime | Yes | 0.21% | NR | NR | Cohort | Treated/ diagnosed |
| Sutterland, 2013 | Europe | The Netherlands | DSM-IV schizophrenia spectrum disorders | 1996–2006 | 10 years |  | 0.35% (0.33%–0.37%) | NR | NR | Cohort | Treated/ diagnosed |
| Sutterland, 2013 | Europe | The Netherlands | DSM-IV schizophrenia | 1996–2006 | 10 years | Yes | 0.20% | NR | NR | Cohort | Treated/ diagnosed |
| Al-Uzri, 2006 | Europe | South Leicestershire, UK | ICD-10 schizophrenia | NR | NR | Yes | 0.19% | NR | NR | Cross-sectional | Treated/ diagnosed |
| Bamrah, 1991 | Europe | Salford, UK | ICD-9 295, 297 (excluding 295.9, 297.9) | 1983 | Point | Yes | 0.59% | NR | NR | Cohort | Treated/ diagnosed |
| Bamrah, 1991 | Europe | Salford, UK | ICD-9 295, 297 (excluding 295.9, 297.9) | 1984 | 12 months | Yes | 0.70% | NR | NR | Cohort | Treated/ diagnosed |
| Bamrah, 1991 | Europe | Salford, UK | ICD-9 295, 297, 298.3, 298.4, 298.8, 298.9 | 1984 | Point |  | 0.63% | NR | NR | Cohort | Treated/ diagnosed |
| Bamrah, 1991 | Europe | Salford, UK | ICD-9 295, 297, 298.3, 298.4, 298.8, 298.9 | 1984 | 12 months |  | 0.75% | NR | NR | Cohort | Treated/ diagnosed |
| Frisher, 2009 | Europe | UK | Schizophrenia, OXMIS/Read | 1996 | 12 months |  | 99.71 per 100,000 PYE | NR | NR | Cohort | Treated/ diagnosed |
| Frisher, 2009 | Europe | UK | Schizophrenia, OXMIS/Read | 1997 | 12 months |  | 103.39 per 100,000 PYE | NR | NR | Cohort | Treated/ diagnosed |
| Frisher, 2009 | Europe | UK | Schizophrenia, OXMIS/Read | 1998 | 12 months |  | 95.44 per 100,000 PYE | NR | NR | Cohort | Treated/ diagnosed |
| Frisher, 2009 | Europe | UK | Schizophrenia, OXMIS/Read | 1999 | 12 months |  | 98.29 per 100,000 PYE | NR | NR | Cohort | Treated/ diagnosed |
| Frisher, 2009 | Europe | UK | Schizophrenia, OXMIS/Read | 2000 | 12 months |  | 89.83 per 100,000 PYE | NR | NR | Cohort | Treated/ diagnosed |
| Frisher, 2009 | Europe | UK | Schizophrenia, OXMIS/Read | 2001 | 12 months |  | 100.79 per 100,000 PYE | NR | NR | Cohort | Treated/ diagnosed |
| Frisher, 2009 | Europe | UK | Schizophrenia, OXMIS/Read | 2002 | 12 months |  | 97.76 per 100,000 PYE | NR | NR | Cohort | Treated/ diagnosed |
| Frisher, 2009 | Europe | UK | Schizophrenia, OXMIS/Read | 2003 | 12 months |  | 86.76 per 100,000 PYE | NR | NR | Cohort | Treated/ diagnosed |
| Frisher, 2009 | Europe | UK | Schizophrenia, OXMIS/Read | 2004 | 12 months |  | 87.38 per 100,000 PYE | NR | NR | Cohort | Treated/ diagnosed |
| Frisher, 2009 | Europe | UK | Schizophrenia, OXMIS/Read | 2005 | 12 months |  | 74.72 per 100,000 PYE | NR | NR | Cohort | Treated/ diagnosed |
| Goldacre, 1994 | Europe | Oxfordshire, UK | ICD-7 300, ICD-8/ICD-9 295 | 1986 | Point (12 months) | Yes | 0.06% | NR | NR | Cohort | Treated/ diagnosed |
| Goldacre, 1994 | Europe | Oxfordshire, UK | ICD-7 300, ICD-8/ICD-9 295 | 1986 | Point (5 years) | | 0.18% | NR | NR | Cohort | Treated/diagnosed |
| Goldacre, 1994 | Europe | Oxfordshire, UK | ICD-7 300, ICD-8/ICD-9 295 | 1986 | 12 months | Yes | 0.06% | NR | NR | Cohort | Treated/ diagnosed |
| Goldacre, 1994 | Europe | Oxfordshire, UK | ICD-7 300, ICD-8/ICD-9 295 | 1982–1986 | 5 years | Yes | 0.19% | NR | NR | Cohort | Treated/ diagnosed |
| Harvey, 1996 | Europe | Camden, UK | Schizophrenia, schizoaffective psychosis, paranoid psychosis, or possible schizophrenia | 1985–1986 | Point |  | 0.53% | NR | NR | Cross-sectional | Treated/ diagnosed |
| Harvey, 1996 | Europe | Camden, UK | Feighner positive schizophrenia | 1985–1986 | Point |  | 0.31% | NR | NR | Cross-sectional | Treated/ diagnosed |
| Harvey, 1996 | Europe | Camden, UK | DSM-III-R schizophrenia | 1985–1986 | Point | Yes | 0.29% | NR | NR | Cross-sectional | Treated/ diagnosed |
| Harvey, 1996 | Europe | North Camden, UK | Schizophrenia, schizoaffective psychosis, paranoid psychosis, or possible schizophrenia | 1986 | Point |  | 0.47% | NR | NR | Cross-sectional | Treated/ diagnosed |
| Harvey, 1996 | Europe | North Camden, UK | Feighner positive schizophrenia | 1986 | Point |  | 0.26% | NR | NR | Cross-sectional | Treated/ diagnosed |
| Harvey, 1996 | Europe | North Camden, UK | DSM-III-R schizophrenia | 1986 | Point |  | 0.22% | NR | NR | Cross-sectional | Treated/ diagnosed |
| Harvey, 1996 | Europe | South Camden, UK | Schizophrenia, schizoaffective psychosis, paranoid psychosis, or possible schizophrenia | 1985 | Point |  | 0.73% | NR | NR | Cross-sectional | Treated/ diagnosed |
| Harvey, 1996 | Europe | South Camden, UK | Feighner positive schizophrenia | 1985 | Point |  | 0.47% | NR | NR | Cross-sectional | Treated/ diagnosed |
| Harvey, 1996 | Europe | South Camden, UK | DSM-III-R schizophrenia | 1985 | Point |  | 0.49% | NR | NR | Cross-sectional | Treated/ diagnosed |
| Jeffreys, 1997 | Europe | North Camden, UK | Schizophrenia, schizoaffective psychosis, paranoid psychosis, or possible schizophrenia | 1991 | Point |  | 0.51% | 0.59%; 0.80% | Age/morbid risk | Cross-sectional | Treated/ diagnosed |
| Jeffreys, 1997 | Europe | North Camden, UK | Feighner positive schizophrenia | 1991 | Point |  | 0.29% | 0.34%; 0.47% | Age/morbid risk | Cross-sectional | Treated/ diagnosed |
| Jeffreys, 1997 | Europe | North Camden, UK | DSM-III-R schizophrenia | 1991 | Point | Yes | 0.30% | 0.35%; 0.48% | Age/morbid risk | Cross-sectional | Treated/ diagnosed |
| McCreadie, 1997 | Europe | Nithsdale; Nunhead; Norwood, UK | ICD-9 schizophrenia | 1991–1993 | 12 months |  | 0.33% | NR | NR | Cross-sectional | Treated/ diagnosed |
| McCreadie, 1997 | Europe | Nithsdale; Nunhead; Norwood, UK | DSM-III-R schizophrenia, via OCRIT | 1991–1993 | 12 months | Yes | 0.26% | NR | NR | Cross-sectional | Treated/ diagnosed |
| McCreadie, 1997 | Europe | Nithsdale; Nunhead; Norwood, UK | ICD-10 schizophrenia, via OCRIT | 1991–1993 | 12 months |  | 0.30% | NR | NR | Cross-sectional | Treated/ diagnosed |
| McCreadie, 1997 | Europe | Nithsdale, UK | ICD-9 schizophrenia | 1992–1993 | 12 months |  | 0.28% | NR | NR | Cross-sectional | Treated/ diagnosed |
| McCreadie, 1997 | Europe | Nithsdale, UK | DSM-III-R schizophrenia, via OCRIT | 1992–1993 | 12 months |  | 0.24% | NR | NR | Cross-sectional | Treated/ diagnosed |
| McCreadie, 1997 | Europe | Nithsdale, UK | ICD-10 schizophrenia, via OCRIT | 1992–1993 | 12 months |  | 0.24% | NR | NR | Cross-sectional | Treated/ diagnosed |
| McCreadie, 1997 | Europe | Nunhead, UK | ICD-9 schizophrenia | 1991–1992 | 12 months |  | 0.45% | NR | NR | Cross-sectional | Treated/ diagnosed |
| McCreadie, 1997 | Europe | Nunhead, UK | DSM-III-R schizophrenia, via OCRIT | 1991–1992 | 12 months |  | 0.29% | NR | NR | Cross-sectional | Treated/ diagnosed |
| McCreadie, 1997 | Europe | Nunhead, UK | ICD-10 schizophrenia, via OCRIT | 1991–1992 | 12 months |  | 0.41% | NR | NR | Cross-sectional | Treated/ diagnosed |
| McCreadie, 1997 | Europe | Norwood, UK | ICD-9 schizophrenia | 1991–1992 | 12 months |  | 0.30% | NR | NR | Cross-sectional | Treated/ diagnosed |
| McCreadie, 1997 | Europe | Norwood, UK | DSM-III-R schizophrenia, via OCRIT | 1991–1992 | 12 months |  | 0.25% | NR | NR | Cross-sectional | Treated/ diagnosed |
| McCreadie, 1997 | Europe | Norwood, UK | ICD-10 schizophrenia, via OCRIT | 1991–1992 | 12 months |  | 0.26% | NR | NR | Cross-sectional | Treated/ diagnosed |
| Najim, 2013 | Europe | Maidstone, UK | OPCRIT schizophrenia only | NR | NR | Yes | 0.17% | NR | NR | Cross-sectional | Treated/ diagnosed |
| Najim, 2013 | Europe | Maidstone, UK | OPCRIT schizophrenia or schizoaffective disorder | NR | NR |  | 0.23% | NR | NR | Cross-sectional | Treated/ diagnosed |
| Shivashankar, 2013 | Europe | Nithsdale, UK | ICD-10 schizophrenia | 2006 | Point | Yes | 0.36% | NR | NR | Cross-sectional | Treated/ diagnosed |
| Brown, 2004 | North America | Alameda County, US | DSM-IV schizophrenia | 1981–1997 | 17 years | Yes | 0.31%–0.41% | NR | NR | Cohort | Treated/ diagnosed |
| Brown, 2004 | North America | Alameda County, US | DSM-IV schizophrenia or schizoaffective disorder | 1981–1997 | 17 years |  | 0.48%–0.54% | NR | NR | Cohort | Treated/ diagnosed |
| Brown, 2004 | North America | Alameda County, US | DSM-IV schizophrenia spectrum disorder | 1981–1997 | 17 years |  | 0.59% | NR | NR | Cohort | Treated/ diagnosed |
| Desai, 2013 | North America | US | ICD-9 295 or 298 | 2005–2008 | 12 months | Yes | 0.25% | NR | NR | Cohort | Treated/ diagnosed |
| Kendler, 1996 | North America | US | Schizophrenia or schizophreniform disorder, via CIDI | 1990–1992 | Lifetime |  | NR | 1.3% (SE: 0.2) | Sampling bias | Cross-sectional | Overall |
| Kendler, 1996 | North America | US | Schizophrenia, via CIDI | 1990–1992 | Lifetime |  | NR | 1.1% (SE: 0.2) | Sampling bias | Cross-sectional | Overall |
| Kendler, 1996 | North America | US | DSM-III-R schizophrenia or schizophreniform disorder | 1990–1992 | Lifetime |  | NR | 0.16% (SE: 0.06) | Sampling bias | Cross-sectional | Overall |
| Kendler, 1996 | North America | US | DSM-III-R schizophrenia | 1990–1992 | Lifetime | Yes | NR | 0.15% (SE: 0.05) | Sampling bias | Cross-sectional | Overall |
| Wu, 2006 | North America | US | ICD-9 295 | 2002 | 12 months | Yes | NR | 0.51%–0.53% | Insurance coverage type | Cohort | Treated/ diagnosed |
| Nuevo, 2012 | All regions | Total (52 countries) | Schizophrenia, self-report | 2003 | Lifetime | Yes | NR | 1.11% (SE: 0.05) | Age, gender | Cross-sectional | Treated/ diagnosed |
| Nuevo, 2012 | Europe | Croatia | Schizophrenia, self-report | 2003 | Lifetime |  | NR | 1.97% (SE: 0.50) | Age, gender | Cross-sectional | Treated/ diagnosed |
| Nuevo, 2012 | Europe | Czech Republic | Schizophrenia, self-report | 2003 | Lifetime |  | NR | 0.37% (SE: 0.18) | Age, gender | Cross-sectional | Treated/ diagnosed |
| Nuevo, 2012 | Europe | Estonia | Schizophrenia, self-report | 2003 | Lifetime |  | NR | 1.50% (SE: 0.42) | Age, gender | Cross-sectional | Treated/ diagnosed |
| Nuevo, 2012 | Europe | Hungary | Schizophrenia, self-report | 2003 | Lifetime |  | NR | 2.56% (SE: 0.54) | Age, gender | Cross-sectional | Treated/ diagnosed |
| Nuevo, 2012 | Europe | Latvia | Schizophrenia, self-report | 2003 | Lifetime |  | NR | 0.93% (SE: 0.50) | Age, gender | Cross-sectional | Treated/ diagnosed |
| Nuevo, 2012 | Asia | Malaysia | Schizophrenia, self-report | 2003 | Lifetime |  | NR | 0.24% (SE: 0.06) | Age, gender | Cross-sectional | Treated/ diagnosed |
| Nuevo, 2012 | Africa | Mauritius | Schizophrenia, self-report | 2003 | Lifetime |  | NR | 0.67% (SE: 0.16) | Age, gender | Cross-sectional | Treated/ diagnosed |
| Nuevo, 2012 | Central/South America | Mexico | Schizophrenia, self-report | 2003 | Lifetime |  | NR | 0.36% (SE: 0.04) | Age, gender | Cross-sectional | Treated/ diagnosed |
| Nuevo, 2012 | Europe | Russian Federation | Schizophrenia, self-report | 2003 | Lifetime |  | NR | 0.41% (SE: 0.12) | Age, gender | Cross-sectional | Treated/ diagnosed |
| Nuevo, 2012 | Europe | Slovakia | Schizophrenia, self-report | 2003 | Lifetime |  | NR | 0.25% (SE: 0.14) | Age, gender | Cross-sectional | Treated/ diagnosed |
| Nuevo, 2012 | Europe | Slovenia | Schizophrenia, self-report | 2003 | Lifetime |  | NR | 1.08% (SE: 0.45) | Age, gender | Cross-sectional | Treated/ diagnosed |
| Nuevo, 2012 | Africa | South Africa | Schizophrenia, self-report | 2003 | Lifetime |  | NR | 1.21% (SE: 0.29) | Age, gender | Cross-sectional | Treated/ diagnosed |
| Nuevo, 2012 | Europe | Spain | Schizophrenia, self-report | 2003 | Lifetime |  | NR | 0.49% (SE: 0.13) | Age, gender | Cross-sectional | Treated/ diagnosed |
| Nuevo, 2012 | Asia | United Arab Emirates | Schizophrenia, self-report | 2003 | Lifetime |  | NR | 1.51% (SE: 0.66) | Age, gender | Cross-sectional | Treated/ diagnosed |
| Nuevo, 2012 | Central/South America | Uruguay | Schizophrenia, self-report | 2003 | Lifetime |  | NR | 0.75% (SE: 0.11) | Age, gender | Cross-sectional | Treated/ diagnosed |
| Nuevo, 2012 | NA | Total for World Bank category high/upper-mid (15 countries) | Schizophrenia, self-report | 2003 | Lifetime |  | NR | 1.00% (SE: 0.10) | Age, gender | Cross-sectional | Treated/ diagnosed |
| Nuevo, 2012 | Asia | Bangladesh | Schizophrenia, self-report | 2003 | Lifetime |  | NR | 0.78% (SE: 0.20) | Age, gender | Cross-sectional | Treated/ diagnosed |
| Nuevo, 2012 | Europe | Bosnia and Herzegovina | Schizophrenia, self-report | 2003 | Lifetime |  | NR | 0.10% (SE: 0.06) | Age, gender | Cross-sectional | Treated/ diagnosed |
| Nuevo, 2012 | Central/South America | Brazil | Schizophrenia, self-report | 2003 | Lifetime |  | NR | 1.67% (SE: 0.22) | Age, gender | Cross-sectional | Treated/ diagnosed |
| Nuevo, 2012 | Africa | Burkina Faso | Schizophrenia, self-report | 2003 | Lifetime |  | NR | 1.30% (SE: 0.27) | Age, gender | Cross-sectional | Treated/ diagnosed |
| Nuevo, 2012 | Africa | Chad | Schizophrenia, self-report | 2003 | Lifetime |  | NR | 3.15% (SE: 0.41) | Age, gender | Cross-sectional | Treated/ diagnosed |
| Nuevo, 2012 | Asia | China | Schizophrenia, self-report | 2003 | Lifetime |  | NR | 0.27% (SE: 0.08) | Age, gender | Cross-sectional | Treated/ diagnosed |
| Nuevo, 2012 | Africa | Comoros | Schizophrenia, self-report | 2003 | Lifetime |  | NR | 0.80% (SE: 0.28) | Age, gender | Cross-sectional | Treated/ diagnosed |
| Nuevo, 2012 | Africa | Congo | Schizophrenia, self-report | 2003 | Lifetime |  | NR | 3.91% (SE: 1.10) | Age, gender | Cross-sectional | Treated/ diagnosed |
| Nuevo, 2012 | Africa | Cote d’Ivoire | Schizophrenia, self-report | 2003 | Lifetime |  | NR | 1.17% (SE: 0.29) | Age, gender | Cross-sectional | Treated/ diagnosed |
| Nuevo, 2012 | Central/South America | Dominican Republic | Schizophrenia, self-report | 2003 | Lifetime |  | NR | 1.23% (SE: 0.16) | Age, gender | Cross-sectional | Treated/ diagnosed |
| Nuevo, 2012 | Central/South America | Ecuador | Schizophrenia, self-report | 2003 | Lifetime |  | NR | 0.94% (SE: 0.21) | Age, gender | Cross-sectional | Treated/ diagnosed |
| Nuevo, 2012 | Africa | Ethiopia | Schizophrenia, self-report | 2003 | Lifetime |  | NR | 1.45% (SE: 0.22) | Age, gender | Cross-sectional | Treated/ diagnosed |
| Nuevo, 2012 | Asia | Georgia | Schizophrenia, self-report | 2003 | Lifetime |  | NR | 0.51% (SE: 0.17) | Age, gender | Cross-sectional | Treated/ diagnosed |
| Nuevo, 2012 | Africa | Ghana | Schizophrenia, self-report | 2003 | Lifetime |  | NR | 0.64% (SE: 0.14) | Age, gender | Cross-sectional | Treated/ diagnosed |
| Nuevo, 2012 | Central/South America | Guatemala | Schizophrenia, self-report | 2003 | Lifetime |  | NR | 0.38% (SE: 0.09) | Age, gender | Cross-sectional | Treated/ diagnosed |
| Nuevo, 2012 | Asia | India | Schizophrenia, self-report | 2003 | Lifetime |  | NR | 2.51% (SE: 0.30) | Age, gender | Cross-sectional | Treated/ diagnosed |
| Nuevo, 2012 | Asia | Kazakhstan | Schizophrenia, self-report | 2003 | Lifetime |  | NR | 0.45% (SE: 0.13) | Age, gender | Cross-sectional | Treated/ diagnosed |
| Nuevo, 2012 | Africa | Kenya | Schizophrenia, self-report | 2003 | Lifetime |  | NR | 0.73% (SE: 0.21) | Age, gender | Cross-sectional | Treated/ diagnosed |
| Nuevo, 2012 | Asia | Laos | Schizophrenia, self-report | 2003 | Lifetime |  | NR | 0.32% (SE: 0.09) | Age, gender | Cross-sectional | Treated/ diagnosed |
| Nuevo, 2012 | Africa | Malawi | Schizophrenia, self-report | 2003 | Lifetime |  | NR | 1.24% (SE: 0.24) | Age, gender | Cross-sectional | Treated/ diagnosed |
| Nuevo, 2012 | Africa | Mali | Schizophrenia, self-report | 2003 | Lifetime |  | NR | 2.25% (SE: 0.42) | Age, gender | Cross-sectional | Treated/ diagnosed |
| Nuevo, 2012 | Africa | Mauritania | Schizophrenia, self-report | 2003 | Lifetime |  | NR | 2.72% (SE: 0.54) | Age, gender | Cross-sectional | Treated/ diagnosed |
| Nuevo, 2012 | Africa | Morocco | Schizophrenia, self-report | 2003 | Lifetime |  | NR | 0.66% (SE: 0.22) | Age, gender | Cross-sectional | Treated/ diagnosed |
| Nuevo, 2012 | Asia | Myanmar | Schizophrenia, self-report | 2003 | Lifetime |  | NR | 0.33% (SE: 0.12) | Age, gender | Cross-sectional | Treated/ diagnosed |
| Nuevo, 2012 | Africa | Namibia | Schizophrenia, self-report | 2003 | Lifetime |  | NR | 2.99% (SE: 0.48) | Age, gender | Cross-sectional | Treated/ diagnosed |
| Nuevo, 2012 | Asia | Nepal | Schizophrenia, self-report | 2003 | Lifetime |  | NR | 2.54% (SE: 0.31) | Age, gender | Cross-sectional | Treated/ diagnosed |
| Nuevo, 2012 | Asia | Pakistan | Schizophrenia, self-report | 2003 | Lifetime |  | NR | 1.12% (SE: 0.19) | Age, gender | Cross-sectional | Treated/ diagnosed |
| Nuevo, 2012 | Central/South America | Paraguay | Schizophrenia, self-report | 2003 | Lifetime |  | NR | 0.46% (SE: 0.10) | Age, gender | Cross-sectional | Treated/ diagnosed |
| Nuevo, 2012 | Asia | Philippines | Schizophrenia, self-report | 2003 | Lifetime |  | NR | 0.43% (SE: 0.08) | Age, gender | Cross-sectional | Treated/ diagnosed |
| Nuevo, 2012 | Africa | Senegal | Schizophrenia, self-report | 2003 | Lifetime |  | NR | 1.48% (SE: 0.35) | Age, gender | Cross-sectional | Treated/ diagnosed |
| Nuevo, 2012 | Asia | Sri Lanka | Schizophrenia, self-report | 2003 | Lifetime |  | NR | 0.65% (SE: 0.16) | Age, gender | Cross-sectional | Treated/ diagnosed |
| Nuevo, 2012 | Africa | Swaziland | Schizophrenia, self-report | 2003 | Lifetime |  | NR | 5.70% (SE: 0.73) | Age, gender | Cross-sectional | Treated/ diagnosed |
| Nuevo, 2012 | Africa | Tunisia | Schizophrenia, self-report | 2003 | Lifetime |  | NR | 1.84% (SE: 0.25) | Age, gender | Cross-sectional | Treated/ diagnosed |
| Nuevo, 2012 | Europe | Ukraine | Schizophrenia, self-report | 2003 | Lifetime |  | NR | 0.60% (SE: 0.15) | Age, gender | Cross-sectional | Treated/ diagnosed |
| Nuevo, 2012 | Asia | Vietnam | Schizophrenia, self-report | 2003 | Lifetime |  | NR | 0.07% (SE: 0.04) | Age, gender | Cross-sectional | Treated/ diagnosed |
| Nuevo, 2012 | Africa | Zambia | Schizophrenia, self-report | 2003 | Lifetime |  | NR | 0.72% (SE: 0.15) | Age, gender | Cross-sectional | Treated/ diagnosed |
| Nuevo, 2012 | Africa | Zimbabwe | Schizophrenia, self-report | 2003 | Lifetime |  | NR | 1.07% (SE: 0.23) | Age, gender | Cross-sectional | Treated/ diagnosed |
| Nuevo, 2012 | NA | Total for World Bank category low/lower-mid (37 countries) | Schizophrenia, self-report | 2003 | Lifetime |  | NR | 1.38% (SE: 0.06) | Age, gender | Cross-sectional | Treated/ diagnosed |
